# Breast cancer chemotherapy induces vascular dysfunction and hypertension through a NOX4-dependent mechanism

**DOI:** 10.1172/JCI149117

**Published:** 2022-07-01

**Authors:** Piotr Szczepaniak, Mateusz Siedlinski, Diana Hodorowicz-Zaniewska, Ryszard Nosalski, Tomasz P. Mikolajczyk, Aneta M. Dobosz, Anna Dikalova, Sergey Dikalov, Joanna Streb, Katarzyna Gara, Pawel Basta, Jaroslaw Krolczyk, Joanna Sulicka-Grodzicka, Ewelina Jozefczuk, Anna Dziewulska, Blessy Saju, Iwona Laksa, Wei Chen, John Dormer, Maciej Tomaszewski, Pasquale Maffia, Marta Czesnikiewicz-Guzik, Filippo Crea, Agnieszka Dobrzyn, Javid Moslehi, Tomasz Grodzicki, David G. Harrison, Tomasz J. Guzik

**Affiliations:** 1 Department of Medicine, Jagiellonian University Medical College, Krakow, Poland.; 2 Institute of Cardiovascular and Medical Sciences, University of Glasgow, Glasgow, United Kingdom.; 3 Centre for Cardiovascular Science, Queen’s Medical Research Institute, University of Edinburgh, Edinburgh, United Kingdom.; 4 Breast Unit, Department of Surgery, Jagiellonian University Hospital, Krakow, Poland.; 5 Laboratory of Cell Signaling and Metabolic Disorders, Nencki Institute of Experimental Biology of Polish Academy of Sciences, Warsaw, Poland.; 6 Division of Clinical Pharmacology, Department of Medicine, Vanderbilt University, Nashville, Tennessee, USA.; 7 Department of Oncology,; 8 Department of Gynecology and Oncology,; 9 Department of Internal Medicine and Gerontology and; 10 Department of Rheumatology, Jagiellonian University Medical College, Krakow, Poland.; 11 Department of Cellular Pathology, University Hospitals of Leicester, Leicester, United Kingdom.; 12 Division of Cardiovascular Sciences, Faculty of Medicine, Biology and Health, University of Manchester, Manchester, United Kingdom.; 13 Division of Medicine and Manchester Academic Health Science Centre, Manchester University NHS Foundation Trust Manchester, Manchester, United Kingdom.; 14 Institute of Infection, Immunity and Inflammation, University of Glasgow, Glasgow, United Kingdom.; 15 Department of Pharmacy, School of Medicine and Surgery, University of Naples Federico II, Naples, Italy.; 16 Department of Periodontology, Preventive Dentistry and Oral Pathology, Jagiellonian University Medical College, Krakow, Poland.; 17 Department of Periodontology and Oral Sciences Research Group, University of Glasgow Dental School, Glasgow, United Kingdom.; 18 Department of Cardiovascular and Thoracic Sciences, Catholic University of the Sacred Heart, Largo A. Gemelli, Rome, Italy.; 19 Section of Cardio-Oncology and Immunology, Division of Cardiology and the Cardiovascular Research Institute, UCSF, San Francisco, California, USA.

**Keywords:** Vascular Biology, Breast cancer, Cardiovascular disease, Molecular biology

## Abstract

Cardiovascular disease is the major cause of morbidity and mortality in breast cancer survivors. Chemotherapy contributes to this risk. We aimed to define the mechanisms of long-term vascular dysfunction caused by neoadjuvant chemotherapy (NACT) and identify novel therapeutic targets. We studied arteries from postmenopausal women who had undergone breast cancer treatment using docetaxel, doxorubicin, and cyclophosphamide (NACT) and from women with no history of such treatment matched for key clinical parameters. We explored mechanisms in WT and *Nox4^–/–^* mice and in human microvascular endothelial cells. Endothelium-dependent, NO-mediated vasodilatation was severely impaired in patients after NACT, while endothelium-independent responses remained normal. This was mimicked by a 24-hour exposure of arteries to NACT agents ex vivo. When applied individually, only docetaxel impaired endothelial function in human vessels. Mechanistic studies showed that NACT increased inhibitory eNOS phosphorylation of threonine 495 in a Rho-associated protein kinase–dependent (ROCK-dependent) manner and augmented vascular superoxide and hydrogen peroxide production and NADPH oxidase activity. Docetaxel increased expression of the NADPH oxidase NOX4 in endothelial and smooth muscle cells and NOX2 in the endothelium. A NOX4 increase in human arteries may be mediated epigenetically by diminished DNA methylation of the NOX4 promoter. Docetaxel induced endothelial dysfunction and hypertension in mice, and these were prevented in *Nox4^–/–^* mice and by pharmacological inhibition of Nox4 or Rock. Commonly used chemotherapeutic agents and, in particular, docetaxel alter vascular function by promoting the inhibitory phosphorylation of eNOS and enhancing ROS production by NADPH oxidases.

## Graphical abstract

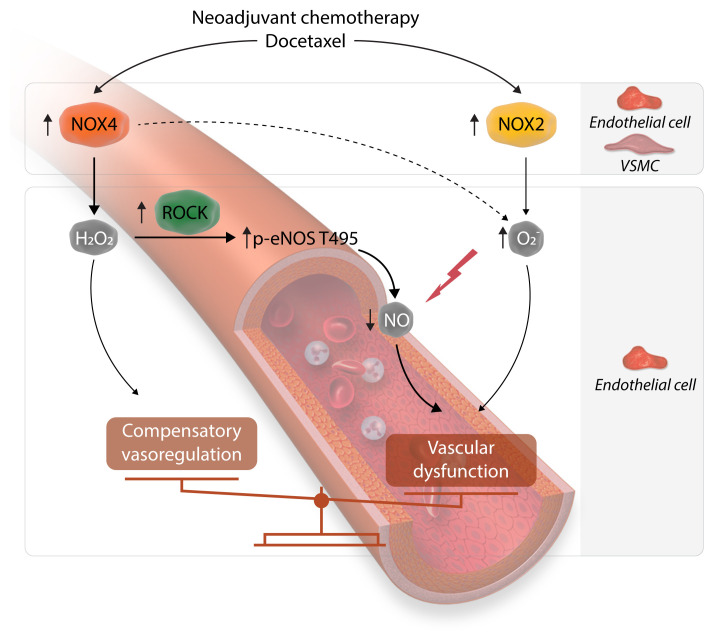

## Introduction

Improvements in breast cancer detection and therapy have significantly increased survival. Breast cancer mortality decreased by 40% between 1989 and 2017 (1). As a result, millions of women alive today are breast cancer survivors. Despite this markedly improved survival, breast cancer survivors are at high risk for atherosclerotic cardiovascular disease (CVD) (2–4). In particular, among women with lower grades of breast cancer, the likelihood of dying from CVD is substantially higher than that of either cancer death (5, 6) or breast cancer recurrence (7). Vascular disease underlies most of these late comorbidities in cancer survivors (5, 6, 8). A multi-hit hypothesis has been proposed to explain this, including risk factors at the time of diagnosis, lifestyle alterations caused by the disease, and the toxicity of drugs and radiation (9). To date, much attention has been focused on anthracycline analogs, which have both immediate and delayed effects on cardiac function (2, 3, 10, 11). Other agents probably also contribute to cardiovascular morbidity in this population. Treatment with tamoxifen increases the risk of myocardial infarction by 1.7-fold (12). Radiation is also known to enhance coronary atherosclerosis, probably via the induction of endothelial injury (9, 13, 14). Given these direct and indirect influences, there is a pressing need to improve cardiovascular outcomes in this patient population.

A standard chemotherapy regimen, often used with surgery, includes taxanes, doxorubicin, and cyclophosphamide (15). Such adjuvant chemotherapy has reduced breast cancer mortality by approximately 15% (16). Given that these agents are commonly used in this population of post-menopausal women, at high risk for cardiovascular disease, it is imperative that we understand their impact on vascular health (17). In the current study, we show that vascular function was markedly impaired in patients who had received docetaxel, doxorubicin, and cyclophosphamide. We found that this impairment was primarily due to docetaxel and was associated with a shift in the balance between the vascular NO bioavailability and ROS production. The latter seems to result from elevated expression and activation of the NADPH oxidase catalytic subunits NOX2 and NOX4. These effects are partially dependent on increased Rho-associated protein kinase (ROCK) activation. Genetic or pharmacological inhibition of murine Nox4 prevents the development of vascular dysfunction, implicating NOX4 as a target in preventing the vascular consequences of chemotherapy.

## Results

### Chemotherapy induces endothelial dysfunction in human arteries.

**Figure 1:**
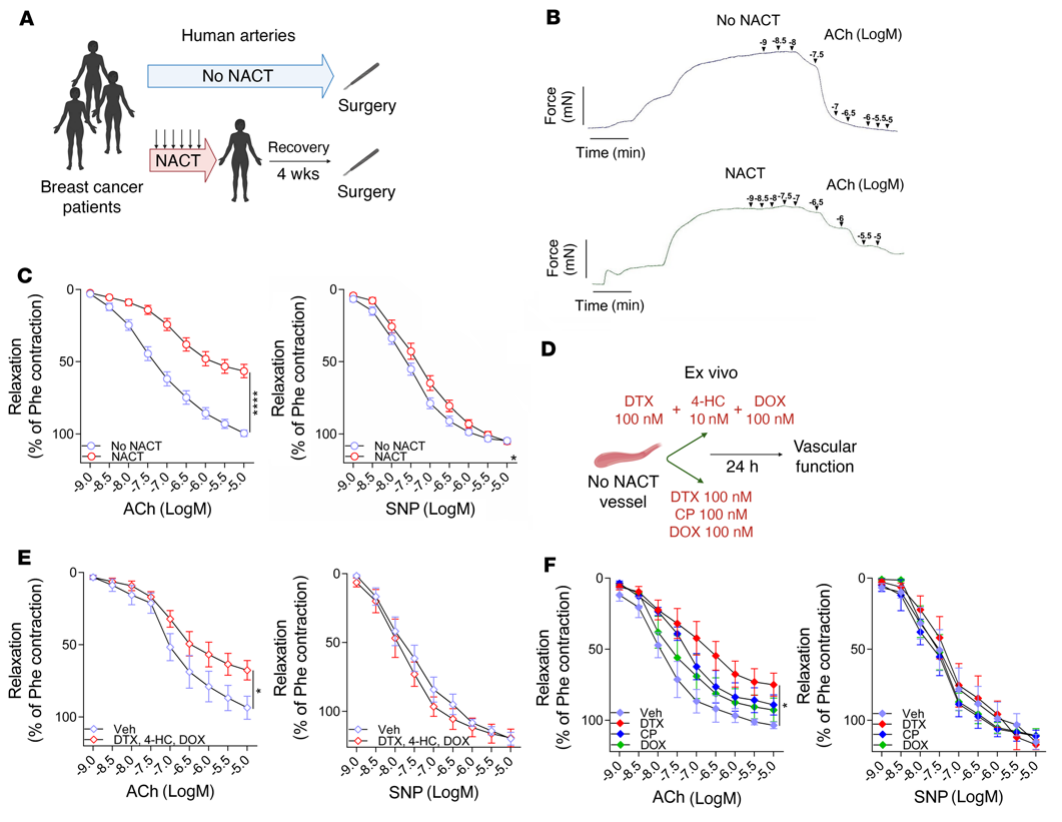
Effects of breast cancer neoadjuvant chemotherapy using docetaxel, cyclophosphamide, and doxorubicin (NACT) on vascular function. (**A**) Study design of human blood vessel collection in patients with breast cancer without NACT (No NACT) or after 6 ± 3 courses of NACT prior to surgery. (**B**) Representative examples of endothelium-dependent vasorelaxations in response to increasing concentrations of ACh (1 nM–10 μM) in arteries from patients with or without NACT. (**C**) Average endothelium-dependent vasorelaxation responses to ACh (1 nM–10 μM) and endothelium-independent relaxation responses to SNP (1 nM–10 μM) in arteries from patients without NACT (*n =* 55) and with NACT (*n =* 40). Two rings were studied per patient, and the values were averaged. Data are expressed as the mean ± SEM. ****P < 0.0001 versus no NACT (**C**, left); *P < 0.05 versus no NACT (**C**, right); 2-way, repeated-measures ANOVA. (**D**) Experimental design of an ex vivo organ culture study of the effects of NACT on vascular function in NACT-naive arteries. (**E**) Endothelium-dependent (ACh) and endothelium-independent (SNP) vasorelaxations in NACT-naive arteries after a 24-hour organ culture with the combined NACT components docetaxel (100 nM), 4-hydroperoxycyclophosphamide (4-HC) (10 nM), and doxorubicin (100 nM) or vehicle (Veh, solvent) (paired arterial rings for each treatment; *n* = 7 patients). *P < 0.05 versus vehicle; 2-way, repeated-measures ANOVA. (**F**) Effects of individual components of NACT on endothelium-dependent (ACh) and endothelium-independent (SNP) vasorelaxations in NACT-naive arteries after a 24-hour organ culture with either docetaxel (100 nM), cyclophosphamide (100 nM), doxorubicin (100 nM), or vehicle (solvent) (paired vessel rings for each treatment; *n* = 5). *P < 0.05 versus vehicle; 2-way, repeated-measures ANOVA with Tukey’s test. Data in **E** and **F** are expressed as the mean ± SEM. DTX, docetaxel; DOX, doxorubicin; CP, cyclophosphamide; Phe, phenylephrine.

In initial experiments, we studied arteries from healthy tissue margins that were unaffected by cancer and extracted during breast cancer surgery. Arteries, with a diameter of approximately 1 mm were obtained from 40 postmenopausal women who had received neoadjuvant chemotherapy (NACT) before the surgery and 55 who had not received such treatment (controls, no NACT). Arteries were collected 1 month after the last dose of NACT. These patients did not differ with respect to other demographics or major risk factors known to affect vascular disease, pharmacotherapies, or radiotherapy, apart from the stage of their cancer (Table 1), and there was no difference in blood vessel size or morphology between the 2 groups (Supplemental Figure 1A; supplemental material available online with this article; https://doi.org/10.1172/JCI149117DS1). We used isometric tension studies to assess endothelium-dependent and endothelium-independent vasodilatation in response to acetylcholine (ACh) and sodium nitroprusside (SNP), respectively. We found that NACT significantly altered endothelium-dependent relaxation response to ACh, while having minimal effect on endothelium-independent response to SNP (Figure 1C). Importantly, these changes were observed 1 month after the last cycle of chemotherapy (Figure 1, A and B). NACT-associated endothelial dysfunction was not dependent on the cancer stage, as it was observed in a subset of patients with stage 2 and 3 as well as in those with stage 3 breast cancer (Supplemental Figure 1B). Moreover, the cancer stage was not associated with differences in endothelial function when analyzed separately within the NACT and no-NACT groups (Supplemental Figure 1, C and D).

In additional experiments, we established that a 24-hour ex vivo exposure of human arteries to a combination of docetaxel, doxorubicin, and cyclophosphamide (Figure 1D) also impaired ACh-induced relaxations, while not altering responses to SNP (Figure 1E). Using this ex vivo approach, we found that, individually, doxorubicin and cyclophosphamide did not significantly affect responses to either ACh or SNP, however, exposure to docetaxel alone significantly impaired ACh-mediated vasodilatation but did not alter SNP-evoked relaxations (Figure 1F and Supplemental Table 1).

### Effect of NACT on eNOS expression and phosphorylation.

**Figure 2:**
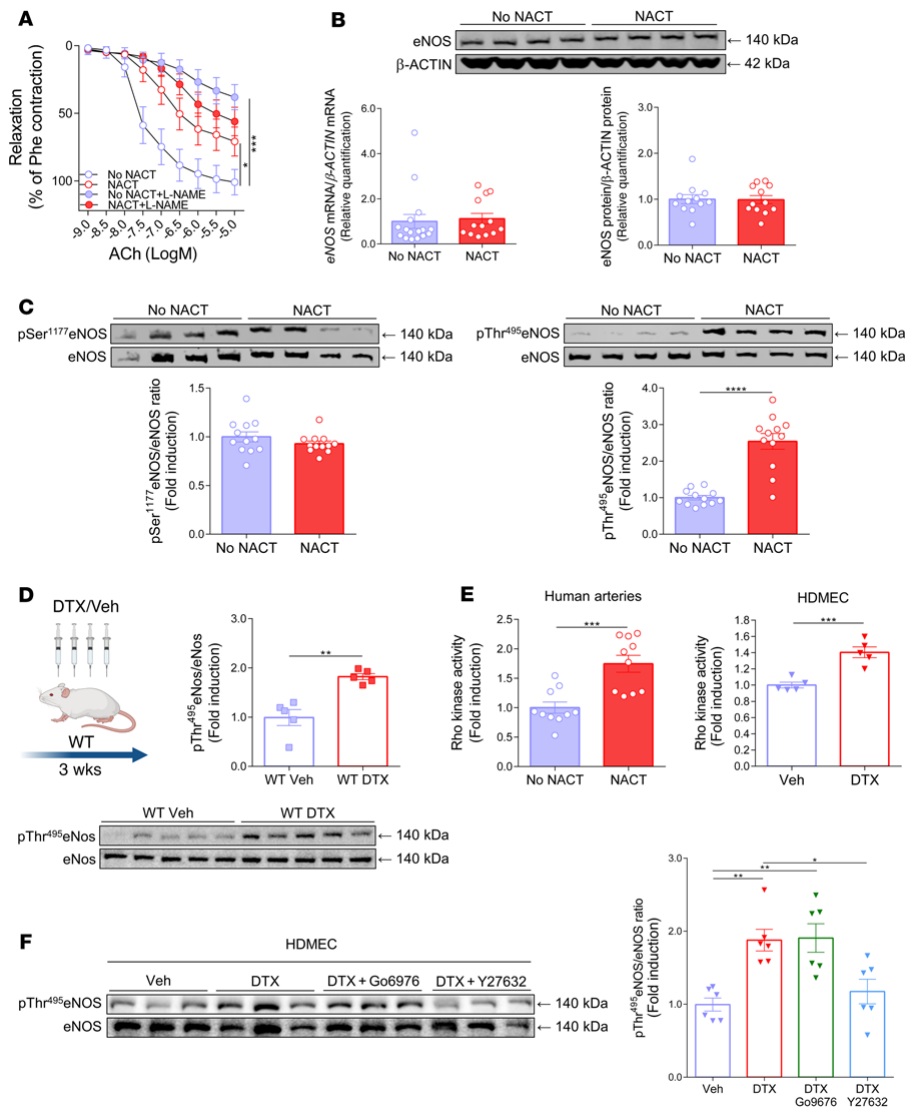
Effects of breast cancer neoadjuvant chemotherapy using docetaxel, cyclophosphamide, and doxorubicin (NACT) on the regulation of eNOS. (**A**) Endothelium-dependent vasorelaxation responses to ACh (1 nM to 10 μM) in arteries from patients without NACT and from patients with prior NACT after preincubation with l-NAME (100 μM) (*n =* 8/group). Data expressed as the mean ± SEM. ***P < 0.001 versus no NACT; *P < 0.05 versus no NACT; 2-way, repeated-measures ANOVA with Tukey’s test. (**B**) Expression of *eNOS* mRNA (*n =* 13–16/group; bottom left) and eNOS protein (Western blotting; *n =* 12/group) in arteries from patients without NACT and from patients who underwent NACT. (**C**) Phosphorylation of eNOS at Ser1177 (left; *n =* 12/group) and at Thr495 in arteries from patients with or without prior NACT (right; *n =* 12/group), normalized to total eNOS. Data were derived from 3 independent experiments and are expressed as the mean ± SEM. ****P < 0.0001 versus no NACT; 2-tailed, unpaired Student’s *t* test. (**D**) Effects of in vivo exposure to docetaxel in C57BL/6J mice (i.p. injections; 10 mg/kg or placebo every 5 days for 3 weeks) on the inhibitory phosphorylation of eNos at Thr495, normalized to total eNOS in mouse aortas (*n =* 5 mice/group). Data are expressed as the mean ± SEM. **P < 0.01 versus WT; 2-tailed, unpaired Student’s *t* test. (**E**) Rho kinase activity in arteries from patients with or without NACT (*n =* 10/group; left) and in HDMECs (*n =* 5/group; right). Data are expressed as the mean ± SEM. ***P < 0.001 versus vehicle or no NACT; 2-tailed, unpaired Student’s *t* test. (**F**) Effects of docetaxel (100 nM) on eNOS at Thr495 phosphorylation in HDMECs and the modulating effect of docetaxel plus Go6976 (1 μM) or docetaxel plus Y27632 (5 μM) (*n =* 6/group). Densitometric analysis of proteins normalized to total eNOS is shown. Immunoblots are from 1 of 2 independent experiments (left) and are expressed as the mean ± SEM. **P < 0.01 versus vehicle or docetaxel; *P < 0.05 versus docetaxel; 1-way ANOVA with Tukey’s test.

In human arteries, endothelium-dependent relaxations can be mediated by NO, vasodilator prostaglandins, and/or endothelium-derived hyperpolarizing factors such as hydrogen peroxide (H_2_O_2_). To define the relative role of NO, we exposed human arteries to the NO synthase inhibitor Nω-nitro-l-arginine methyl ester hydrochloride (l-NAME) before administration of ACh. l-NAME markedly inhibited the endothelium-dependent vasodilatation response to ACh in arteries from women who had not received NACT, while it had a minimal effect on these responses in the arteries of women who had received NACT. This strongly suggests that the NO-mediated mechanisms of endothelium-dependent vasodilatation are impaired by NACT treatment (Figure 2A). As the morphological presence of the endothelial cell layer did not differ between the study groups (Supplemental Figure 1A), reduced endothelial NO synthase (eNOS) expression could provide a potential explanation for endothelial dysfunction. However, we found that NACT was not associated with loss of eNOS mRNA or protein expression (Figure 2B). eNOS is regulated by phosphorylation, predominantly at serine 1177 (Ser1177), which is stimulatory, and at threonine 495 (Thr495), which is inhibitory. As shown in Figure 2C, there was no change in phosphorylation of Ser1177, but there was an increase in the inhibitory phosphorylation of eNOS at Thr495. This observation was confirmed in vivo in mice exposed to docetaxel for 3 weeks via i.p. injections every 5 days (Figure 2D) and in vitro in cultured human dermal microvascular endothelial cells (HDMECs) (Figure 2E), supporting the hypothesis that eNOS phosphorylation at Thr 495 is a crucial mechanism of impaired vascular function in patients with prior NACT.

Thr495 can be phosphorylated by protein kinase C (PKC) and Rho kinase (ROCK) (18). Rho kinase activity was induced in human arteries after NACT, and docetaxel increased the Rho kinase activity in HDMECs (Figure 2E). To further explore the role of ROCK and PKC, we exposed HDMECs in culture to docetaxel with and without the PKC inhibitor Go6976 or the Rho kinase inhibitor Y27632. As shown in Figure 2F and Supplemental Figure 2A, ex vivo exposure of cultured endothelial cells and human vessel organ culture to docetaxel enhanced the phosphorylation of eNOS on Thr495, mimicking the in vivo effect of NACT. This was not changed by inhibiting PKC, but was markedly reduced by the Rho kinase inhibitor. Thus, inhibitory phosphorylation of eNOS via Rho kinase probably contributes to the loss of endothelium-dependent vasodilatation following exposure to docetaxel. Rho kinase activity was induced by NACT in human arteries, indicating its potential role in endothelial dysfunction. Moreover, docetaxel increased Rho kinase activity in HDMECs (Figure 2E). In addition, the Rho kinase inhibitor Y27632 markedly improved endothelium-dependent relaxation responses to ACh in the arteries of patients who had previously undergone NACT (Supplemental Figure 2B).

Importantly, the mechanisms regulating eNOS activity dependence on the binding of HSP90α to eNOS were not changed in HDMECs exposed to docetaxel (Supplemental Figure 2C), and HSP90α expression was unaltered in NACT-exposed human arteries (Supplemental Figure 1D).

### Docetaxel modulates key pathways relevant to vascular function.

**Figure 3:**
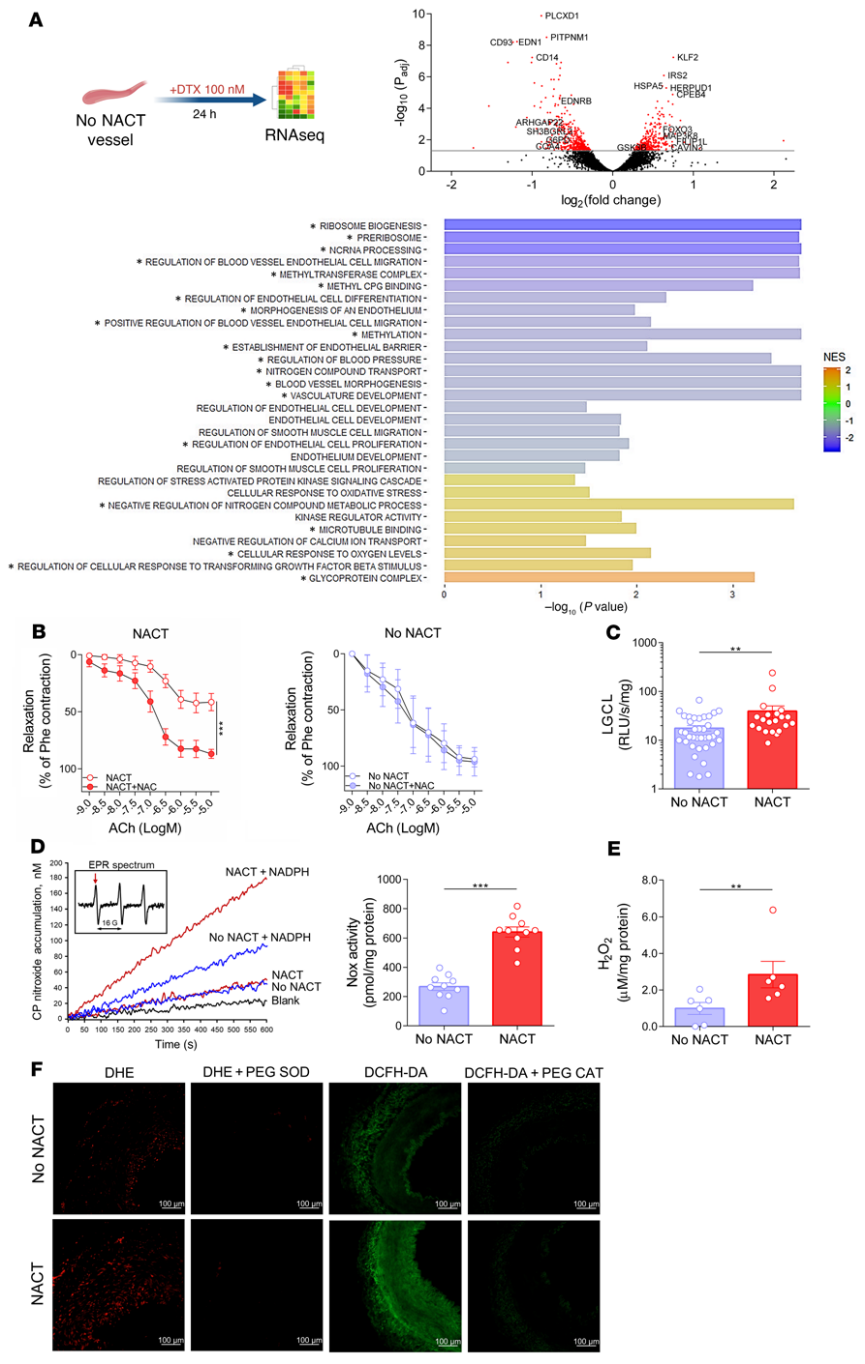
Vascular effects of docetaxel and the role of oxidative stress in NACT-induced endothelial dysfunction. (**A**) Schematic design of ex vivo organ culture study of the effects of 24 hours of docetaxel on the gene expression profile using RNA-Seq. Volcano plot shows examples of significantly altered genes related to vascular biology and oxidative stress in NACT-naive arteries after a 24-hour organ culture with docetaxel (100 nM) in comparison with exposure to vehicle (red: adjusted *P* value [*P*_adj_] < 0.05). Bar charts present the GSEA (–log_10_ *P* values) of selected docetaxel-downregulated (blue) and -upregulated (yellow) pathways (*n =* 6/group). *P_adj_ < 0.05, FDR. Enrichment is expressed as the normalized enrichment score (NES). (**B**) Effect of NAC (1 mM) on endothelium-dependent vasorelaxation responses to ACh in arteries from patients with or without prior neoadjuvant chemotherapy (NACT versus no NACT; *n =* 5/group). Data are expressed as the mean ± SEM. ***P < 0.001 versus NACT; 2-way, repeated-measures ANOVA. (**C**) Superoxide production was measured using lucigenin (LGCL; 5 μM) in arteries from patients with (*n =* 27) or without (*n =* 37) prior NACT. **P < 0.01 versus no NACT. (**D**) EPR of NADPH-dependent superoxide production in membrane fractions of arteries from patients with or without prior NACT; example time scans of CP-nitroxide accumulation using CPH (1-hydroxy-3-carboxy-2,2,5,-tetramethyl-pyrrolidine hydrochloride; 1.0 mM) and NADPH (0.2 mM). Insert shows the initial EPR spectrum of the spin probe CPH; arrow indicates the low-field component used to follow the nitroxide accumulation. (**D**, right) Data indicate the mean ± SEM of NADPH oxidase (Nox) activity (*n =* 10/group). ***P < 0.001, versus no NACT. (**E**) H_2_O_2_ production using Amplex Red (*n =* 6/group; mean ± SEM). **P < 0.01 versus no NACT; 2-tailed, unpaired Student’s *t* test (**C**–**E**). (**F**) Microphotographs of fluorescence detection of superoxide (DHE; 10 μM) and H_2_O_2_ production (DCFH-DA; 10 μM) in arteries from patients with or without prior NACT. PEG-SOD (500 U/mL) and PEG-CAT (500 U/mL) were used to show signal specificity for superoxide and H_2_O_2_, respectively (representative of 5/group). Scale bars: 100 μm.

To gain further insight into the mechanisms responsible for altered endothelium-dependent vasodilatation, we exposed vascular segments to docetaxel or placebo for 24 hours in organ culture experiments and analyzed global RNA expression using RNA-Seq (Figure 3A). We found that the expression of 802 mRNA transcripts was significantly (FDR *P <* 0.05) altered by docetaxel treatment (Figure 3A and Supplemental Table 4), which contributed to significant changes in gene sets and pathways relevant to vascular function. These included endothelial and smooth muscle cellular processes such as differentiation, morphogenesis, migration, and signaling as well as pathways involved in epigenetic regulation and DNA methylation. Importantly, a broad range of vascular processes that were altered were redox sensitive. These processes included endothelial and smooth muscle cell biogenesis, proliferation, and migration, kinase signaling, blood pressure regulation, cellular response to oxidative stress, and oxygen sensing (Figure 3A). This is consistent with the role of ROS in vascular dysfunction in wide range of cardiovascular pathologies (19).

### Role of ROS in altering vascular function in response to NACT.

Increased production of ROS plays a major role in regulating vascular function in pathological states. To investigate the importance of this mechanism, we examined the effect of the antioxidant *N*-acetylcysteine (NAC) on ACh-evoked vascular relaxations. As shown in Figure 3B, NAC markedly improved endothelium-dependent relaxation responses to ACh in arteries of patients who previously received NACT, but had no effect in the non-NACT group (Figure 3B). NAC can react with and scavenge several ROS, including superoxide, H_2_O_2_, and the hydroxyl radical. In additional experiments, we observed increased vascular superoxide production by arteries from women who had received NACT compared with those who had not (Figure 3C).

NADPH oxidases are major sources of ROS in human arteries (19). We therefore used electron spin resonance to examine NADPH oxidase activity in homogenates of arteries from patients who had received NACT and from those who had not and found a significant increase in superoxide production in response to NADPH in those with prior NACT (Figure 3D). Amplex Red assays also demonstrated an increase in vascular production of H_2_O_2_ in arteries of women who had undergone prior NACT (Figure 3E). Using the fluorescent dyes dihydroethidium (DHE) to detect superoxide and 2′,7′-dichlorofluorescein (DCF) to detect H_2_O_2_, we detected the production of both superoxide and H_2_O_2_ predominantly in the media, with a less apparent contribution from the endothelium. These signals were eliminated by the selective superoxide and H_2_O_2_ scavengers PEG superoxide dismutase (SOD) and PEG catalase (PEG-CAT), respectively (Figure 3F and Supplemental Figure 2E).

### Effect of NACT on the vascular expression of NADPH oxidase catalytic subunits.

**Figure 4:**
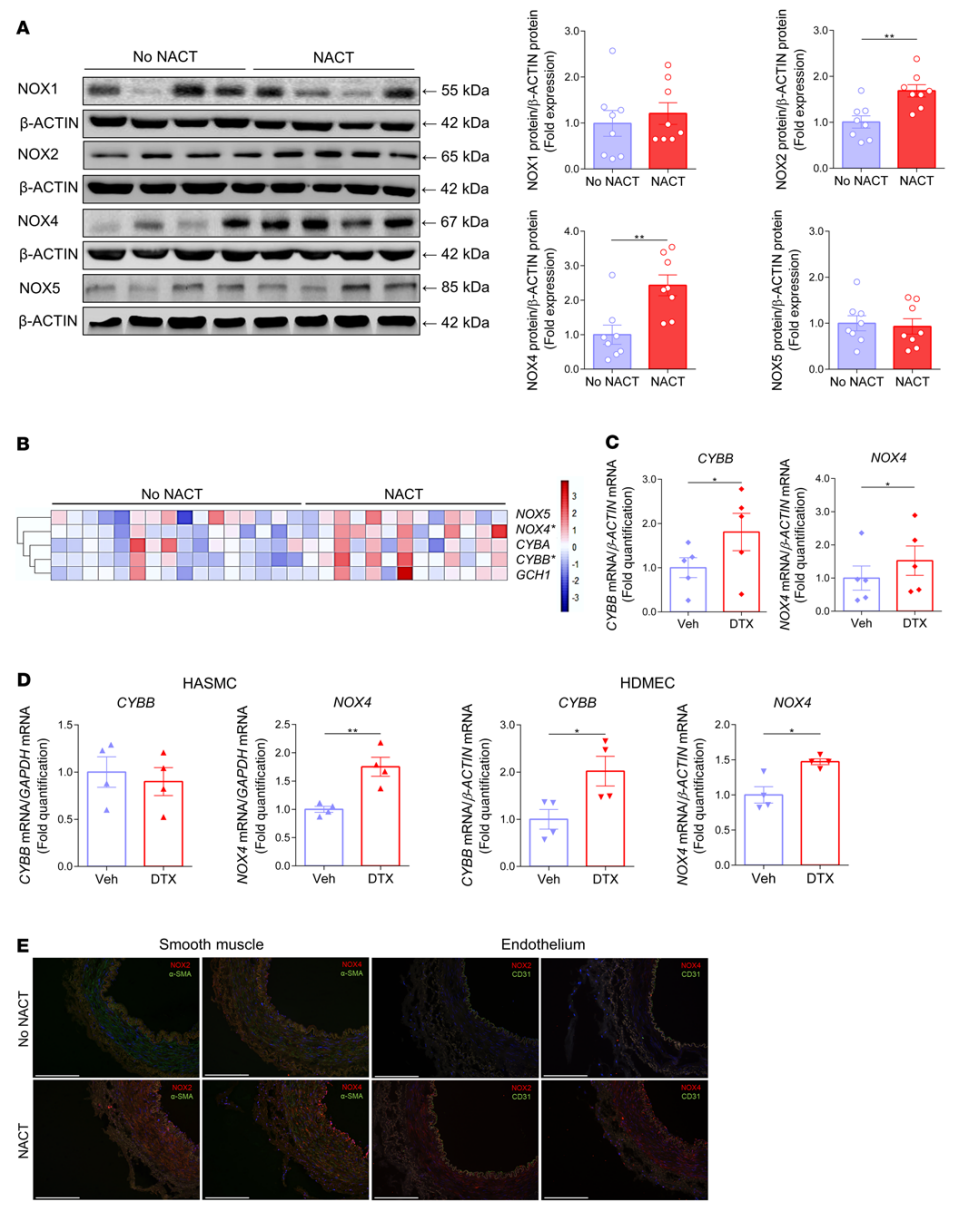
Mechanisms of oxidative stress induced by NACT and docetaxel in the vasculature. (**A**) Protein levels of the NADPH oxidase homologs NOX1, NOX2, NOX4, and NOX5 in arteries from patients with or without prior NACT, with the densitometric analysis normalized to β-ACTIN (right) (*n =* 8/group). Data are expressed as the mean ± SEM. **P < 0.01 versus no NACT; 2-tailed, unpaired Student’s *t* test. (**B**) Heatmap depicting mRNA expression of selected key genes involved in the regulation of oxidative stress in human vasculature in arteries from patients with (*n =* 13) or without (*n =* 16) prior NACT. Expression was normalized to *ACTB* mRNA. *P < 0.05 versus no NACT; 2-tailed, unpaired Student’s *t* test. (**C**) Effects of a 24-hour organ culture with docetaxel (100 nM) or vehicle on the expression of NOX2 (also known as *CYBB*) and *NOX4* mRNA in NACT-naive arteries (*n =* 5/group). Data are expressed as the mean ± SEM. *P < 0.05 versus vehicle; 2-tailed, paired Student’s *t* test. (**D**) Effects of 24 hours of docetaxel exposure (100 nM) on NOX2 (*CYBB*) and *NOX4* mRNA in HASMCs and HDMECs (*n =* 4/group). Data are expressed as the mean ± SEM. **P < 0.01 versus vehicle; *P < 0.05 versus vehicle; 2-tailed, unpaired Student’s *t* test. (**E**) Immunofluorescence detection (red) of NOX2 and NOX4 expression in arteries from patients with or without NACT. Costaining (green) with CD31 and α-SMA was done to identify NOX2 and NOX4 expression in endothelial cells and SMCs (representative of 5/group). Scale bars: 200 μm.

There are 4 key NOX proteins in human vascular cells: NOX1, -2, -4, and -5. Using Western blotting (Figure 4A) and reverse transcription PCR (RT-PCR) (Figure 4B), we found that there was no change in NOX1 or NOX5 expression and increased expression of NOX2 and NOX4 in the arteries of women who had undergone NACT. These increases in NOX2 and NOX4 were recapitulated by a 24-hour exposure of vascular segments to docetaxel (Figure 4C). Through additional experiments using cultured endothelial and vascular smooth muscle cells (VSMCs), we found that docetaxel increased *NOX2* and *NOX4* expression in endothelial cells and *NOX4* expression in smooth muscle cells (Figure 4D). Immunofluorescence showed that NOX2 staining was predominantly increased in the vascular media, whereas NOX4 was increased in both the media and endothelial cells of women who had undergone NACT (Figure 4E).

**Figure 5:**
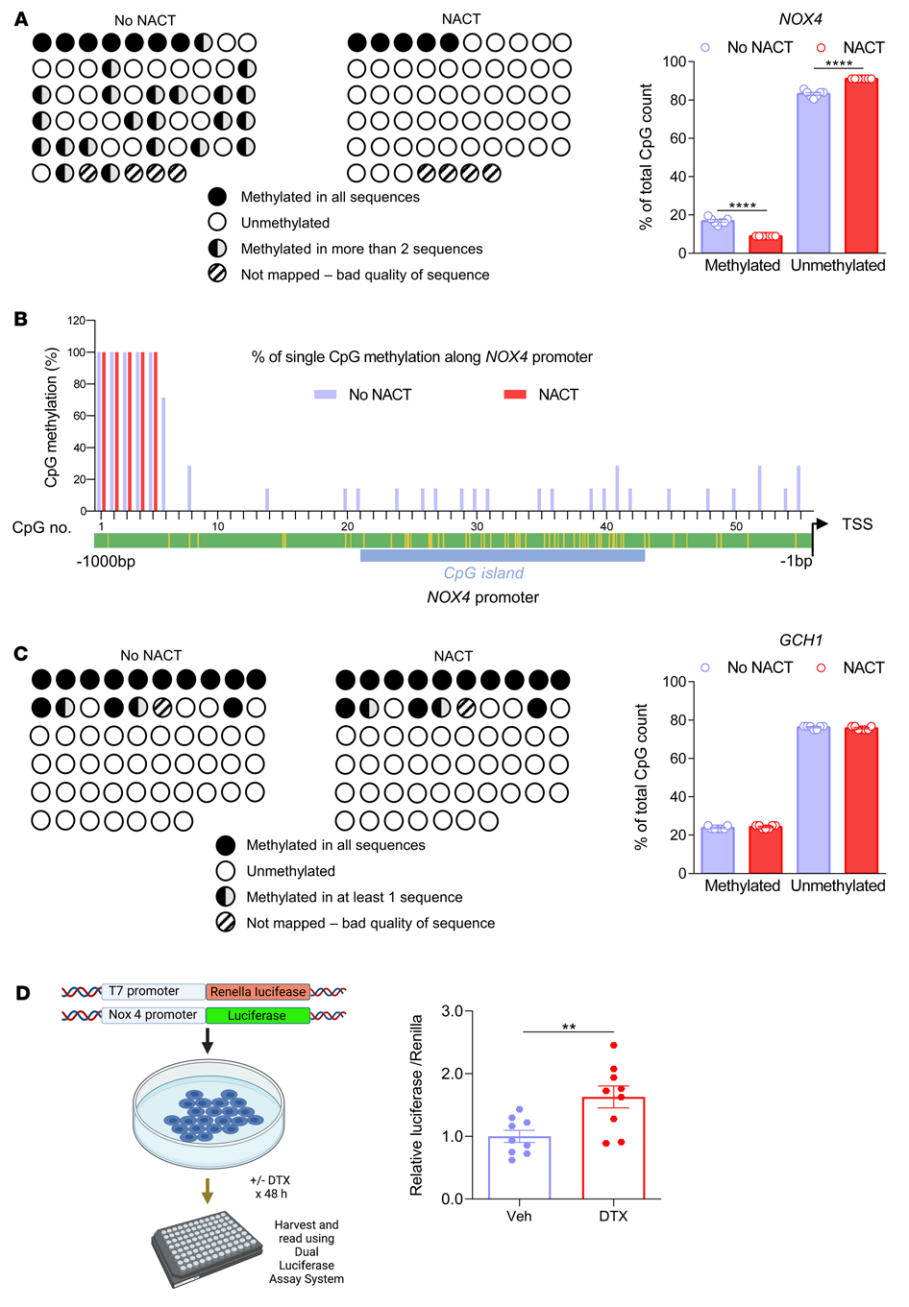
Methylation of the *NOX4* promoter was induced by NACT. (**A**) Bisulfite sequencing analysis of the *NOX4* promoter in arteries from patients with or without prior NACT showing the methylation levels. Schematic representation shows promoter methylation (left) and mean data on the percentage of the total CpG count (right; *n =* 7/group). ****P < 0.0001 versus no NACT; 2-tailed, unpaired Student’s *t* test. (**B**) Percentage of single CpG methylation along the *NOX4* promoter. (**C**) Bisulfite sequencing analysis of the *GCH1* promoter in arteries from patients with or without prior NACT, showing methylation levels. Schematic representation of promoter methylation (upper) and mean data on the percentage of the total CpG count (right, *n =* 7/group). Data were analyzed by 2-tailed, unpaired Student’s *t* test. (**D**) *NOX4* promoter assay in HEK293 cells treated with docetaxel (100 nM) or placebo (*n =* 9/group). Data are expressed as the mean ± SEM. **P < 0.01 versus docetaxel; 2-tailed, unpaired Student’s *t* test.

Considering the prolonged effect of NACT observed 4 weeks after its discontinuation, we considered that epigenetic mechanisms might modulate *NOX4* expression over the long term. Indeed, studies of the *NOX4* promoter showed that 23 CpG sites exhibited decreased DNA methylation in arteries from women that had undergone NACT compared with no-NACT donor arteries (Figure 5, A and B). We found that 6% of the CpG sites located in the CpG island of the *NOX4* promoter were normally methylated in arteries of women who had not received NACT and that NACT therapy eliminated methylation of these CpG sites (Figure 5, A and B, and Supplemental Table 5). As a control, we studied methylation of the GTP cyclohydrolase gene (*GCH1*) and found that it was unaltered by NACT (Figure 5C). To further understand the effects of docetaxel on the *NOX4* promoter, we performed an in vitro promoter activity assay in HEK293 cells. A 48-hour exposure to docetaxel induced *NOX4* promoter activity in an in vitro promoter activity assay in HEK293 cells (Figure 5D).

### Docetaxel induces hypertension and vascular dysfunction in mice via a Nox4-dependent mechanism.

**Figure 6:**
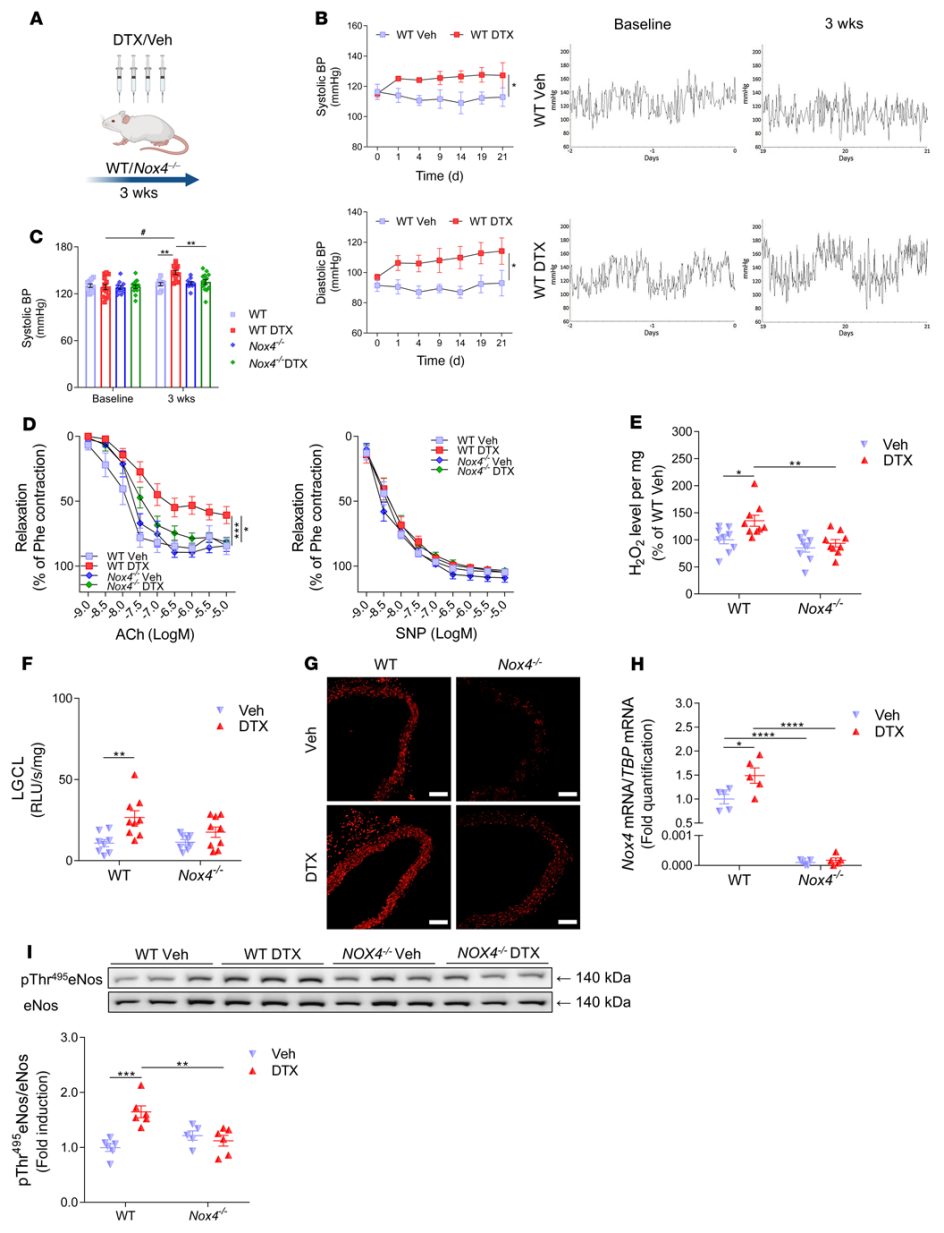
Functional role of Nox4 in the regulation of vascular dysfunction by docetaxel in vivo. (**A**) Schematic design of in vivo studies of the effects of docetaxel on vascular endpoints. *Nox4^–/–^* and WT mice were injected i.p. with docetaxel (10 mg/kg) or placebo (solvent) every 5 days for 3 weeks. (**B**) Effect of docetaxel on blood pressure (BP) in C57BL/6J mice by telemetry. Data are expressed as the mean ± SEM. *P < 0.05 versus WT; 2-way, repeated-measures ANOVA. (**C**) Systolic blood pressure by tail-cuff plethysmography in *Nox4^–/–^* and WT mice treated with docetaxel or placebo (*n =* 10–14/group). Data are expressed as the mean ± SEM. ^#^*P <* 0.01 versus baseline; **P < 0.01 versus WT or docetaxel-treated *Nox4^–/–^* mice; 2-way, repeated-measures ANOVA with Tukey’s test. (**D**) Endothelium-dependent vasorelaxation to ACh (1 nM–10 μM) and endothelium-independent vasorelaxation to SNP (1 nM–10 μM) in mouse aortas (*n =* 9–14/group). Data are expressed as the mean ± SEM. ***P < 0.001 versus WT; *P < 0.05 versus *Nox4^–/–^* mice treated with docetaxel; 2-way, repeated-measures ANOVA with Tukey’s test. (**E**) H_2_O_2_ production using Amplex Red in aortas from *Nox4^–/–^* and WT mice treated with docetaxel or placebo (*n =* 9/group). Data are expressed as the mean ± SEM. **P < 0.01 versus *Nox4^–/–^* mice treated with docetaxel; *P < 0.05 versus WT; 2-way ANOVA with Bonferroni’s test. (**F**) LGCL (5 μM) in aortas (*n =* 8–9/group). Data are expressed as the mean ± SEM. **P < 0.01 versus WT; 2-way ANOVA with Bonferroni’s test. (**G**) Representative images showing superoxide production in mouse aortas using DHE fluorescence. Scale bars: 100 μm. (**H**) *Nox4* mRNA expression in mouse aortas (*n =* 5/group). Data are expressed as the mean ± SEM. ****P < 0.0001 versus *Nox4^–/–^* mice treated with docetaxel; *P < 0.05 versus WT; 2-way ANOVA with Bonferroni’s test. (**I**) p-eNOS (Thr495) in *Nox4^–/–^* and WT mouse aortas treated with docetaxel or placebo (*n =* 5–6/group). Densitometric analysis was normalized to total eNos. Immunoblots represent 1 of 2 independent experiments and are summarized as the mean ± SEM. **P < 0.01 versus *Nox4^–/–^* mice treated with docetaxel; ***P < 0.001 versus WT; 2-way ANOVA and Bonferroni’s test.

To gain additional insight into the role of NOX4 in response to docetaxel, we treated WT and *Nox4^–/–^* mice with this agent (10 mg/kg every 5 days for 3 weeks, Figure 6A). Measurements using radiotelemetry showed that blood pressure significantly increased following the first docetaxel injection and was sustained throughout treatment with this agent (Figure 6B). In mice lacking *Nox4*, this hypertensive response was absent (Figure 6C). In agreement with our findings in human arteries, isometric tension studies of the aorta showed that treatment with docetaxel impaired endothelium-dependent vasorelaxation responses to ACh (Figure 6D) but did not affect endothelium-independent vasodilatation responses to SNP (Figure 6D). To confirm the role of Nox4-derived superoxide in impairing endothelium-dependent vasodilatation, we also treated arteries in the organ chamber with GKT137831, a selective inhibitor of NOX1 and NOX4. This agent normalized ACh-induced vasodilatation in mice previously treated with docetaxel (Supplemental Figure 3G). In line with our results in human arteries, docetaxel treatment increased vascular H_2_O_2_ (Figure 6E) and superoxide (Figure 6F) production in WT mice, and these responses were absent in *Nox4*-deficient mice (Figure 6, E and F). DHE fluorescence confirmed an increase of superoxide production in the media of WT mouse aortas treated with docetaxel that was absent in *Nox4^–/–^* mice (Figure 6G). RT-PCR analysis revealed that aortic *Nox4* mRNA expression was increased in WT mice treated with docetaxel and was absent in the *Nox4^–/–^* mice (Figure 6H).

As shown in Figure 2F, docetaxel treatment promoted ROCK-dependent phosphorylation of eNOS at Thr495. Since H_2_O_2_ can activate Rho kinase, we examined Thr495 phosphorylation on eNOS in WT and *Nox4^–/–^* mice and found that this inhibitory phosphorylation increased by docetaxel treatment in WT but not *Nox4^–/–^* mice (Figure 6I).

Microtubule stabilization is a key mechanism of action of taxanes (20) and is also needed for basal eNOS phosphorylation and subcellular organization (21). One way that taxanes affect microtubule stabilization is the lysine acetylation of α-tubulin (AcK40) (20). We found that lysine acetylation was an unlikely mechanism of docetaxel-induced vascular dysfunction, as docetaxel treatment similarly increased Ack40–α-tubulin in WT and *Nox4^–/–^* mouse aortas, despite striking differences in vascular and blood pressure phenotypes (Supplemental Figure 3H). In line with increased acetylation, however, changes in direct eNOS acetylation cannot be excluded.

**Figure 7:**
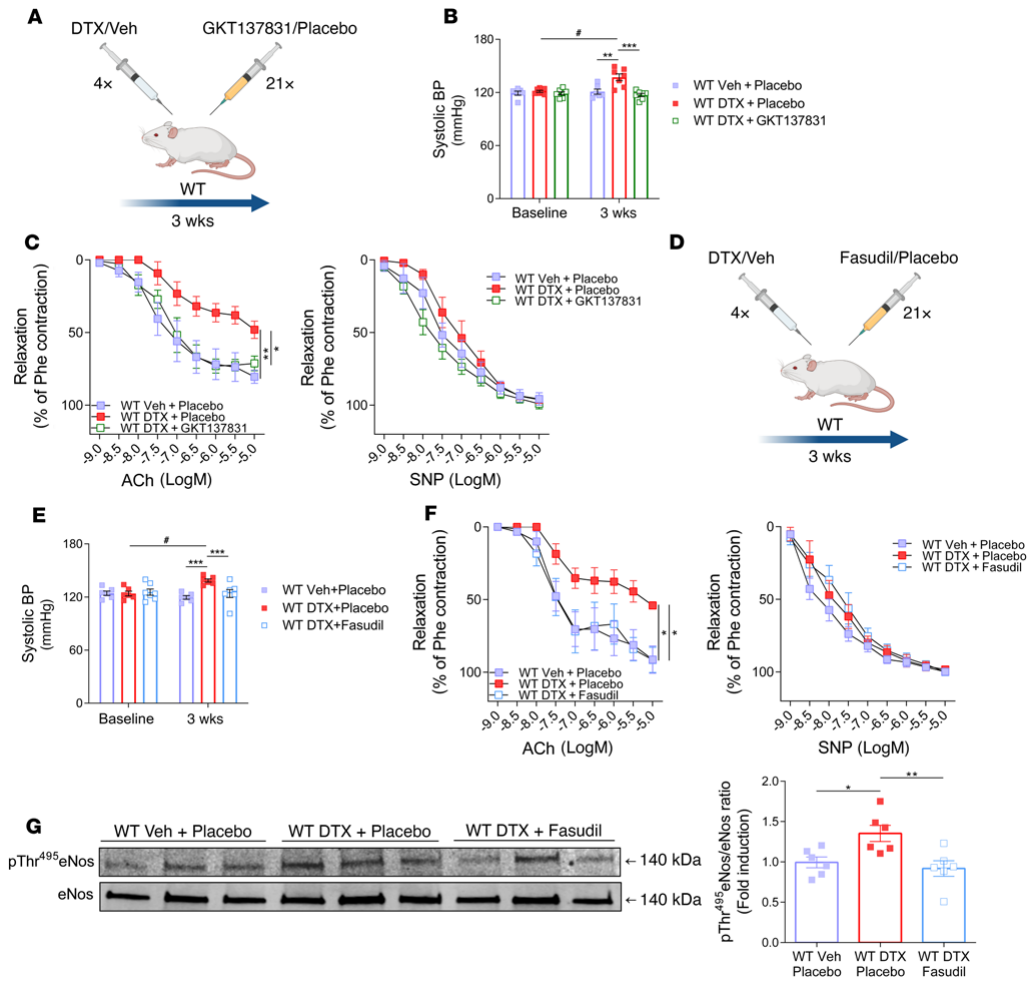
Functional role of Nox4 and Rho kinase in the regulation of vascular dysfunction by docetaxel in vivo. (**A**) Schematic of the study design for WT mice injected with vehicle, docetaxel (10 mg/kg every 5 days), or docetaxel plus the Nox1/4 inhibitor GKT137831 (40 mg/kg every day) or placebo (solvent) for 3 weeks. (**B**) Systolic blood pressure by tail-cuff plethysmography (*n =* 6–7/group). Data are expressed as the mean ± SEM. ^#^*P* < 0.01 versus baseline; **P < 0.01 versus WT (vehicle + placebo); ***P < 0.001 versus WT treated with docetaxel plus GKT137831; 2-way, repeated-measures ANOVA with Tukey’s test. (**C**) Endothelium-dependent vasorelaxation responses to ACh (1 nM–10 μM) and endothelium-independent vasorelaxation responses to SNP (1 nM–10 μM) in mouse aortas (*n =* 5–6/group). Data are expressed as the mean ± SEM. *P < 0.05 versus WT treated with docetaxel plus GKT137831; **P < 0.01 versus WT; 2-way, repeated-measures ANOVA with Tukey’s test. (**D**) Study design, for WT mice injected with docetaxel (10 mg/kg every 5 days) or docetaxel (10 mg/kg every 5 days) and the ROCK inhibitor fasudil (30 mg/kg every day) or placebo (solvent) for 3 weeks. (**E**) BP was measured by tail-cuff plethysmography (*n =* 6–7/group). Data are expressed as the mean ± SEM. ^#^*P* < 0.01 versus baseline; ***P < 0.01 versus WT mice and WT mice treated with docetaxel plus fasudil; 2-way, repeated-measures ANOVA with Tukey’s test. (**F**) Endothelium-dependent vasorelaxation responses to ACh (1 nM–10 μM) and endothelium-independent vasorelaxation responses to SNP (1 nM–10 μM) in mouse aortas (*n =* 6/group). Data are expressed as the mean ± SEM. *P < 0.05 versus WT and WT mice treated with docetaxel plus fasudil; 2-way, repeated-measures ANOVA with Tukey’s test. (**G**) Effect of fasudil on phosphorylation of eNos at Thr495 in WT mice treated with docetaxel (*n =* 6/group). Densitometric analysis of proteins normalized to total eNos expression. Immunoblots are from 1 of 2 independent experiments. Data are expressed as the mean ± SEM. *P < 0.05 versus WT; **P < 0.01 versus WT mice treated with docetaxel plus fasudil; 1-way ANOVA with Tukey’s test.

To confirm that Nox4 represents a therapeutic target for preventing the development of vascular dysfunction, we administered daily i.p. injections of the Nox1/Nox4 inhibitor GKT137831 during docetaxel exposure (Figure 7A) and found that this prevented both the increase in blood pressure (Figure 7B) and development of vascular dysfunction in mouse aortas (Figure 7C) caused by this taxane.

In keeping with our observations in human arteries, ROCK inhibition by fasudil (Figure 7D) prevented the blood pressure increase (Figure 7E) and vascular dysfunction caused by docetaxel (Figure 7F) in mice. Fasudil decreased the docetaxel-induced Thr495 phosphorylation of eNOS (Figure 7G), further supporting the role of ROCK in docetaxel-induced eNOS dysfunction.

Other possible mechanisms of the blood pressure effects of docetaxel in mice were investigated. Docetaxel did not change the heart rate of mice after 3 weeks of treatment (Supplemental Figure 3B). Treatment of mice with docetaxel did not affect aldosterone levels (Supplemental Figure 3C), renal damage as assessed by histopathology (Supplemental Figure 3D), urinary albumin (Supplemental Figure 3E), or urinary neutrophil gelatinase–associated lipocalin (NGAL) (Supplemental Figure 3F).

## Discussion

In the present study, we identify a mechanism by which the commonly used chemotherapeutic agent docetaxel promoted vascular dysfunction and loss of NO-mediated vasodilatation. This was in part mediated by an increase in vascular production of ROS through the NADPH oxidase subunits NOX4 and NOX2. ROS produced by these enzymes likely have 2 major effects. First, superoxide reacts at a diffusion-limited rate with NO, limiting the vasodilatation caused by the latter and yielding the strong prooxidant peroxynitrite (22). Second, H_2_O_2_ stimulates Rho kinase, which in turn causes inhibitory phosphorylation of eNOS (23), reducing its ability to produce NO (18). Both effects appear to play a role in the vascular dysfunction caused by docetaxel.

NACT-induced ROS overproduction was observed in both endothelial cells and smooth muscle cells. Importantly, either short-lived superoxide or more stable H_2_O_2_, produced by VSMCs have been shown to affect endothelial function by direct scavenging of NO and by production of H_2_O_2_, which in turn regulates signaling and gene expression. Our findings were based on an analysis of human arteries, and we gained additional mechanistic insight from studies of WT and *Nox4^–/–^* mice, providing possible mechanisms of long-term vascular disease in breast cancer survivors.

Although we did not directly measure NO release, the loss of NO-dependent vasodilatations was profound. In addition to mediating vasodilatation, NO regulates vascular homeostasis, inhibiting the adhesion of platelets to the endothelium and VSMC proliferation and reducing vascular expression of adhesion molecules and chemokines (21). These effects are antiatherogenic (21), and thus the deleterious effects of docetaxel we observed are likely to promote atherosclerosis and contribute to the increased cardiovascular risk observed in this population.

Our data are in keeping with the in vitro observations of Hung et al., who exposed HUVECs to docetaxel in culture and showed that this increased ROS production and apoptosis through in PKC-dependent fashion (24). These authors implicated both the mitochondria and NADPH oxidase as playing a role in this response (24). In human arteries and cells, we found that PKC inhibition did not affect NO-dependent vasodilatation or eNOS phosphorylation; however, in other studies, we confirmed an increase in the activity of several PKC subunits, consistent with the observations of Hung et al. (24). Others have reported that docetaxel promotes the degradation of HSP90, which is an important chaperone for eNOS (25). However, we did not observe a decrease in HSP90α in arteries following NACT, suggesting that this is an unlikely mechanism of endothelial function in humans treated with this drug (Supplemental Figure 2D). Our findings of increased ROS production and enhanced inhibitory phosphorylation of eNOS provide an alternate mechanism of the long-term vascular effects of docetaxel in vivo. In the long term, such alterations may result in eNOS uncoupling, whereby eNOS contributes to oxidative stress rather than to vasoprotective mechanisms (26).

In addition to causing endothelial dysfunction and oxidative stress, our studies in mice showed that docetaxel caused a sustained elevation of blood pressure. We are unaware of studies showing that this agent causes hypertension in humans, however the accurate clinical assessment of blood pressure is notoriously haphazard, and studies using 24-hour ambulatory monitoring of blood pressure at baseline and following docetaxel therapy are warranted. Importantly, the incidence of hypertension in middle-aged women who may be receiving this agent approaches 50%, and these individuals are often treated with antihypertensive agents (27).

The effects of docetaxel may depend on vascular pathology. For example, it inhibits hypoxia and Sugen-induced pulmonary hypertension in rats (28). This is clearly different from our observation and probably reflects the central role of cell proliferation in pulmonary hypertension (28).

An important finding of our study is that we observed continued endothelial dysfunction and increased ROS production in human arterial segments 1 month after cessation of NACT. This indicates a sustained change in endothelial biology caused by chemotherapy. In keeping with this, we observed a reduction in methylation of the *NOX4* promoter in these blood vessels (29) that may underlie the long-term effect on the expression of this NADPH oxidase catalytic subunit. Potential mechanisms of reduction of NOX4 promoter methylation are not understood, however, several enzymes are involved in DNA demethylation including ten-eleven translocation methylcytosine dioxygenase (TET), thymine-DNA glycosylase (TDG), and lysine-specific demethylase 1 (30). The regulation of these enzymes by docetaxel requires additional study. In addition, their activity may be modulated by oxidative stress in the context of vascular dysfunction. Of interest, DNA methyltransferase (DNMT) activity is associated with resistance to docetaxel (31), providing an additional link.

A possible interpretation of the vascular dysfunction seen in patients receiving NACT might be that they have advanced cancer, which could promote inflammation and oxidative injury (3). Our studies in which we exposed normal human arteries to docetaxel are helpful in this regard, because we found that this agent induced vascular dysfunction, ROS production, and an inhibition of eNOS phosphorylation that was exaggerated compared with that seen in control vascular segments not treated with docetaxel. Moreover, endothelial dysfunction in the studied arteries was not associated with the patient’s cancer stage. Finally, we also found that docetaxel induced vascular abnormalities and hypertension in mice in the absence of cancer. Therefore, it is unlikely that our findings in humans are related to their underlying disease.

Although our study focused on the use of docetaxel, evidence of endothelial dysfunction has been reported in an acute setting in vivo in patients treated with paclitaxel (32). It is therefore likely that docetaxel and paclitaxel, and taxanes in general, share similar mechanisms of action. Thus, our observations may shed light on the clinical observations indicating a possible increase in cardiovascular events following the implantation of paclitaxel-coated stents (33).

Other components of NACT may exert similar molecular effects and contribute to endothelial dysfunction in vivo (34). Direct ex vivo effects of doxorubicin on vascular function have been observed at very high concentrations, probably due to redox cycling. Doxorubicin has been reported to increase ROS production at a concentration of 10 μMol/L, but did not alter endothelial function (35). Nevertheless, the additive effects of docetaxel and doxorubicin on the vasculature may be clinically relevant in patients receiving chemotherapy.

NADPH oxidases are a key source of oxidative stress in human arteries (19). Their expression and activity are increased in the context of traditional risk factors for atherosclerosis such as diabetes or hypercholesterolemia (19). Importantly, the prevalence of traditional risk factors did not differ between patients in our study. These observations suggest that NACT promotes vascular dysfunction at least in part in a manner similar to that of traditional risk factors of atherosclerosis associated with oxidative stress–dependent endothelial dysfunction. Indeed, increased expression and activity of NOX2 are characteristic features of atherosclerosis, diabetes, as well as hypertension (36). Interestingly, the NOX4-dependent effects that underlie docetaxel-induced vascular dysfunction may not mirror the role of this homolog in atherosclerosis (37). Recent studies have shown that NOX4 may prevent the development of atherosclerosis and endothelial dysfunction (38) in apolipoprotein E–deficient (*apoE^–/–^*) mice (39). NOX4 has also been shown to be atheroprotective in diabetes (40). However, in several other studies, NOX4 has been shown to have pathogenic roles in atherosclerosis (41), heart failure (42), and more recently in cognitive impairment and dementia (43). This dual activity of NOX4 is likely associated with its ability to produce H_2_O_2_ rather than superoxide (44). Approximately 90% of the electron flux isolated through Nox4 produces H_2_O_2_, and 10% forms superoxide (45). Through its vasodilatory properties, H_2_O_2_ can provide a compensatory mechanism for maintaining vasomotor function (46), while simultaneously activating a number of proatherosclerotic, redox-sensitive genes and, as demonstrated in the case of docetaxel, to activate Rho kinase–dependent inhibitory phosphorylation of eNOS (47).

Our findings illuminate potential therapeutic avenues for NACT-driven endothelial dysfunction. HMG-CoA (3-hydroxy-3-methyl-glutaryl-coenzyme A) reductase inhibitors (statins) prevent both Rho kinase activation by geranylation of the small G protein Rac and thus NADPH oxidase activation (19). Likewise, the Rho kinase inhibitor fasudil is being studied for Raynaud’s phenomenon and heart failure and might prove useful in preventing inhibitory phosphorylation of eNOS in individuals treated with docetaxel (48). Moreover, observations that increased NOX4 expression is associated with its promoter demethylation suggest that targeting this epigenetic mechanism could harness oxidative stress in the context of docetaxel treatment (49). Finally, NOX1/NOX4 inhibitors such as GKT137831 might prove beneficial in this patient population to reduce cardiovascular complications (50). The latter was demonstrated in human vessel organ cultures as well as in the mouse model, showing the effectiveness of GKT137831 in preventing vascular dysfunction. The safety and efficacy of long-term Nox4 inhibition need to be carefully evaluated and take into consideration the protective effects of Nox4 in atherosclerosis and vascular remodeling (38–40, 51).

In conclusion, we have demonstrated that NACT for breast cancer induces vascular oxidative stress in humans. This is largely due to the effects of docetaxel and is dependent on an increase in the expression of the NOX4 and NOX2 NADPH oxidases. Epigenetic mechanisms may be important in mediating NOX4 upregulation. These mechanisms act in concert to impair endothelial function and promote vascular disease. Our study describes what we believe to be a novel, previously unknown vascular effect of docetaxel and identifies therapeutic targets that may benefit breast cancer survivors who have received neoadjuvant chemotherapy.

## Methods

### Study participants.

Ninety-five women with breast cancer were recruited prior to surgery, including patients who did not receive prior neoadjuvant chemotherapy (no NACT, control group, *n =* 55) and patients who received prior neoadjuvant chemotherapy (NACT, study group, *n =* 40). The patients were recruited in the Department of General, Oncological and Gastroenterological Surgery at the University Hospital in Krakow. The NACT group received 6 ± 3 cycles of NACT consisting of 75 mg/m^2^ body surface area (BSA) of docetaxel, 50 mg/m^2^ BSA of doxorubicin, and 500 mg/m^2^ BSA of cyclophosphamide. The last cycle of NACT was for each patient administered at least 4 weeks before the surgery.

The inclusion criteria were as follows: post-menopausal women (at least 12 months after the last menstruation) with locally advanced breast cancer (without distant metastases).

The exclusion criteria were as follows: (a) cardiovascular diseases including past cardiovascular events, coronary artery disease, heart failure, valvular heart disease, or symptomatic atherosclerosis; (b) chronic inflammatory diseases (bronchial asthma, atopic, rheumatoid, or autoimmune diseases); (c) acute inflammatory disease in the previous month; (d) other cancers and/or a history of chemotherapy; (e) hormone therapy before surgery; (f) previous radiation therapy; and (g) drug or alcohol addiction.

The study was conducted with the approval of the local bioethics committee of Jagiellonian University (KBET/84/B/2009 and 1072.6120.340.2020). Written informed consent was obtained from all patients.

### Human blood vessels.

For all experiments, arteries of approximately 1 mm in diameter, constituting branches of the internal thoracic artery, were isolated from healthy breast tissue obtained during mastectomy surgery. Arteries were immediately transferred to ice-cold Krebs-HEPES buffer and were maintained at 4°C. Surrounding tissues were delicately separated using microsurgical instruments and a microscope, and arteries were then cut into 2 mm rings that were used for subsequent studies. Several steps were performed to reduce bias related to patient-to-patient variability. Patients were selected randomly from medical-surgical oncology lists. In experiments using ex vivo cultures, paired rings from the same patient were used. In these instances, the results from individual rings were averaged and reported as *n =* 1. The selection of arteries for molecular characterization and mechanistic experiments were performed randomly by investigators blinded to the patients’ comorbidities. Blood vessels were studied in random order. Arteries from control and NACT patients were always studied on the same day to minimize experimental variability. N values always reflect the number of individual patients.

### Ex vivo incubation.

Arterial rings from no-NACT patients were incubated with the individual components of NACT: docetaxel, doxorubicin, cyclophosphamide, 4-hydroperoxycyclophosphamide (all from Cayman Chemicals), and vehicle (solvent) as a control. The drug concentrations used were 10 nM or 100 nM. Arteries were incubated in a buffer containing HBSS (Gibco, Thermo Fisher Scientific), 20 mM HEPES (Gibco, Thermo Fisher Scientific), and gentamicin with glutamine in an incubator at 37°C in a humidified atmosphere for 24 hours (52). After incubation, vascular function was measured, or rings were placed in RNAlater (Ambion) for gene expression and RNA-Seq studies.

### In vitro endothelial cell culturing.

Primary HDMECs were acquired from PromoCell. Cells were cultured in the presence of Endothelial Cell Growth Medium MV (PromoCell) with the addition of a 1% mixture of penicillin and streptomycin (Thermo Fisher Scientific). After an initial 4 hours of withdrawal of growth supplements from the culture, cells were incubated with 100 nM docetaxel or 100 nM docetaxel plus 1 μM Go6976 or 100 nM docetaxel plus 5 μM Y27632 (Cayman Chemicals) or vehicle (solvent) for 24 hours at 37°C in a humidified atmosphere of 5% CO_2_.

### In vitro smooth muscle cell cultures.

Primary human aortic smooth muscle cells (HASMCs) were acquired from Thermo Fisher Scientific and cultured in Medium 231 (Thermo Fisher Scientific) supplemented with 20% Smooth Muscle Growth Supplement (SMGS) (Thermo Fisher Scientific) with the addition of a 1% mixture of penicillin and streptomycin (Thermo Fisher Scientific). After 4 hours of starvation, cells were incubated with 100 nM docetaxel (Cayman Chemicals) and vehicle (solvent) for 24 hours at 37°C in a humidified atmosphere of 5% CO_2_.

### In vivo study and treatment.

Studies of mice were conducted according to the ARRIVE (Animal Research: Reporting of In Vivo Experiments) guidelines. We used male C57BL/6J and *Nox4^–/–^* (B6.129-Nox4tm1Kkr/J) mice obtained from The Jackson Laboratory between the ages of 3.5 and 4.5 months. Mice were housed in the animal facility at Jagiellonian University Medical College and at the Faculty of Biochemistry, Biophysics and Biotechnology (Jagiellonian University).

Mice were treated with docetaxel (10 mg/kg, Cayman Chemicals) or placebo (solvent) i.p. every 5 days for 3 weeks. GKT137831 (40 mg/kg, MedChemExpress) or fasudil (30 mg/kg; MedChemExpress) was injected s.c. daily during docetaxel treatment. After this, mice were euthanized using CO_2_ inhalation, and left ventricle perfusion was performed using cold PBS. Aortic rings were used to measure vascular function, ROS generation, and gene and protein expression.

### Blood pressure monitoring in mice.

Blood pressure and heart rate were measured using radiotelemetry as previously described (53). Mouse anesthesia was induced with 3%–5% isoflurane and maintained with 1.5%–3% of this agent. Radiotelemetry units (Data Sciences International) were implanted via a right flank incision and mid-anterior neck incisions. The telemetry catheter was inserted through the right carotid artery and advanced so that the tip was positioned in the aortic arch. One week later, measurements were begun and obtained every 5 minutes throughout the experiment. In some experiments, blood pressure was also measured noninvasively using the Visitech BP 2000 Blood Pressure Analysis System and was monitored every 2 days (54). Animals were placed on a platform heated to 37°C, and blood pressure measurement was performed using the cuff attached to the tail. Mice were acclimated to this procedure by 10 training measurements. These were followed by 15 actual measurements during each session. Pressure measurement was always carried out at the same time of day by an investigator blinded to group allocation.

### Histology.

Arteries frozen in Tissue-Tek OCT were cut at 8 μm with a cryostat and stained with H&E to compare morphological characteristics of the arteries from both groups of patients (55). Formalin-fixed and paraffin-embedded sections of mouse kidney were deparaffinized and rehydrated. Renal histology was performed using a Periodic Acid–Schiff (PAS) Staining kit (MilliporeSigma). An Evos light microscope (Invitrogen, Thermo Fisher Scientific) was used for visualization. Morphological data were evaluated using ImageJ (NIH).

### Vascular function studies.

Human arteries and mouse aortas were cut into 2 mm rings and mounted in an organ bath system (Danish Myo Technology 750TOBS or 610M myograph) (56). Following preconstriction with 120 mM KCl, arteries were constricted with phenylephrine (up to 60%–70% of maximal determined constriction) (MilliporeSigma), and vasorelaxant responses to increasing doses of Ach (1 nM to 10 μM, MilliporeSigma) and SNP (1 nM–10 μM, MilliporeSigma) were examined. Endothelium-dependent vasorelaxation in response to ACh was also studied in the presence of NAC (1 mM), l-NAME (100 μM, MilliporeSigma), Y27632 (10 μM, Cayman Chemical), or GKT137831 (10 μM, Cayman Chemical).

### Western blotting.

Blood vessel segments and HDMECs were homogenized in lysis buffer (1.0% Nonidet P-40, 0.5% sodium deoxycholate, 150 mmol/L NaCl, 1.0 mmol/L EDTA, 0.1% SDS, 2.0 mmol/L sodium orthovanadate [Na3VO4], 1.0 mM PMSF) containing the following inhibitors of proteolytic enzymes: aprotinin (10 μg/mL), leupeptin (10 μg/mL), and pepstatin (10 μg/mL) (MilliporeSigma). The protein concentration in lysates was determined using the DC Assay Protein kit (Bio-Rad). Protein (30 μg) separation was carried out using polyacrylamide gels (7.5%–10%), and proteins were transferred onto a nitrocellulose membrane (Whatman) at 4°C. Nonspecific binding sites on the membrane were blocked with 5% skim milk or 3% BSA in Tris-buffered saline solution with Tween-20 (TBST) for 1 hour at room temperature, and then membranes were incubated with anti–β-actin (Abcam, ab8226, 1:10,000), anti–phosphorylated eNOS (p-eNOS) (Thr495) (BD Biosciences, 612707, 1:500); anti–p-eNOS (Ser1177) (BD Biosciences, 612393, 1:500); anti-eNOS (BD Biosciences, 610297, 1:500); anti-NOX1 (MilliporeSigma, HPA035299, 1:1000); anti-NOX2 (Abcam, ab129068, 1:1000); anti-NOX4 (Abcam, ab109225, 1:1000); anti-NOX5 (MilliporeSigma, SAB2501641, 1:1000); anti–α-tubulin (Cell Signaling Technology, 2125S, 1:1000); anti–acetyl α-tubulin (Cell Signaling Technology, 5335S, 1:1000); anti–γ-tubulin (MilliporeSigma, T6557, 1:1000); and anti-HSP90α (BD Biosciences, 610419, 1:1000) (see also Supplemental Table 2) overnight at 4°C. The next day, membranes were incubated with secondary antibodies conjugated to HRP or with secondary antibodies conjugated to fluorescent dyes (IRDye680 LT or IRDye800 CW, LI-COR). Pierce ECL Western Blotting Substrate (Thermo Fisher Scientific) was used for chemiluminescence-based detection. Final scans were performed using the Azure c500 Western Blot Imaging System (Azure Biosystem) or the Odyssey FC Imaging System (LI-COR). Densitometric analyses were performed using ImageJ or ImageStudio (LI-COR) software.

### RT-PCR.

Isolated blood vessels were preserved in an RNAlater solution (Ambion). RNA isolation was performed using the RNAeasy Mini Kit (QIAGEN). Reverse transcription reactions were performed using Applied Biosystems’ High-Capacity cDNA Reverse Transcription Kit. Quantitative real-time PCR reactions were performed using the 7900HT instrument (Applied Biosystems), TaqMan Gene Expression Assays, and TaqMan Gene Expression Master Mix (Thermo Fisher Scientific) according to the manufacturer’s protocol. Results were analyzed using RQ Manager 1.2.1 and then normalized to the expression level of the housekeeping gene (57).

### RNA-Seq.

RNA was isolated from arterial rings from patients in the no-NACT group and incubated with docetaxel (100 nM) or vehicle (solvent) as a control for 24 hours at 37°C. RNA was isolated using the Direct-Zol RNA MiniPrep kit (Zymo Research) and treated with DNase I. RNA-Seq profiling was performed by GENEWIZ (United Kingdom) (58). Sequence reads were trimmed using Trimmomatic, version 0.36. The trimmed reads were mapped to the *Homo sapiens* GRCh38 reference genome available on ENSEMBL using the STAR aligner, version 2.5.2b, and BAM files were generated. Unique gene hit counts were calculated using featureCounts from the Subread package, version 1.5.2.

### Lucigenin enhanced chemiluminescence.

The level of vascular superoxide production was determined by lucigenin-dependent chemiluminescence (LGLC) (56). Vessels were cut to expose the endothelium and then aerated with 95% O_2_ and 5% CO_2_ in Krebs-HEPES buffer for 20 minutes. Measurements were performed with 5 μM lucigenin (MilliporeSigma) in Krebs-HEPES buffer using a FB12 chemiluminometer (Berthold). ROS production was given in RLU per second per milligram of the dry weight of the vessel.

### Electron paramagnetic resonance.

Superoxide production was additionally measured by electron paramagnetic resonance (EPR) (59). EPR spectra were recorded using a Bruker EMX spectrometer. The measurement of NADPH oxidase vascular activity was performed using a CPH spin probe (1-hydroxy-3-carboxy-2,2,5,5-tetramethylpyrrolidine) and then normalized to the protein content determined by the bicinchoninic acid (BCA) method. The measurement was made in the presence or absence of NADPH (0.2 mM).

### DHE localization of superoxide.

Vascular sections (30 μm thick) were incubated with 10 μM DHE (Molecular Probes) solution for 30 minutes at 37°C in a humidified chamber that was protected from light (60). Vascular superoxide production was also studied in the presence of 500 U/mL PEG-SOD (MilliporeSigma). Fluorescence was excited at 488 nm, and emission was recorded at 605 nm.

### Amplex Red–based measurement.

Protein lysates were incubated with 50 μL of a solution containing 100 μM Amplex Red, 0.2 U/mL HRP, and 1× Reaction Buffer (Invitrogen, Thermo Fisher Scientific) (61). H_2_O_2_ produced by the vessels was quantified by absorbance at 560 nm. The level of H_2_O_2_ was normalized to the protein concentration using the DC Assay Protein kit (Bio-Rad).

Murine aortic H_2_O_2_ production was also determined using 50 μM Amplex Red in the presence of 0.1 U/mL HRP and Krebs-HEPES. Fluorescence was excited at 560 nm and emission recorded at 590 nm. The H_2_O_2_ level was normalized to the dry weight of the aorta.

### DCFH-DA assay.

Arterial sections (30 μm thick) were incubated with a 10 μM DCFH-DA (Molecular Probes) solution for 30 minutes at 37°C and 5% CO_2_ in a humidified chamber, that was protected from light. H_2_O_2_ production was also studied in the presence of 500 U/mL PEG-CAT (MilliporeSigma). Fluorescence was excited at 504 nm, and emission was recorded at 525 nm.

### Immunofluorescence.

Arteries stored in Tissue-Tek OCT were cut into 8 μm sections and placed on slides (62). Nonspecific binding sites were blocked with Protein Block (Dako) containing 0.5% Triton for 1 hour at room temperature and then incubated with primary antibodies overnight at 4°C. The vessels were incubated with anti-NOX2 (Abcam, ab129068); anti-NOX4 (Abcam, ab109225); anti-CD31 (Abcam, ab24590); and anti–α–smooth muscle actin (anti–α-SMA) (MilliporeSigma, A5228) (Supplemental Table 2) overnight at 4°C in a humidified chamber, protected from light. Proteins were detected with the secondary antibodies Alexa Fluor 594 and Alexa Fluor 488 (Life Technologies, Thermo Fisher Scientific) in the dark for 45 minutes. The location of the cell nuclei was detected with DAPI dye (Life Technologies, Thermo Fisher Scientific).

### Methylation studies.

Methylation of the *NOX4* gene promoter was analyzed using bisulfite conversion, cloning, and sequencing. *GCH1* was used as a control gene for these studies. Briefly, genomic DNA was isolated from the arteries using the AllPrep DNA/RNA Mini Kit (QIAGEN). To identify 5-methylcytosine at single bp resolution, purified genomic DNA was subjected to bisulfite conversion using the innuCONVERT Bisulfite All-In-One Kit (Analytik Jena) according to the manufacturer’s protocol. Identification of promoter regions and the transcription start sites (TSSs) of the genes, CpG island prediction, and design of methylation-specific primers were performed (63). The DNA fragments encoding *NOX4* and *GCH1* promoter sequences were amplified on a bisulfite-converted genomic DNA template with Phusion U Hot Start DNA Polymerase (Thermo Fisher Scientific) in a heated-lid thermocycler under the following conditions: 98°C for 30 seconds; 35 × (98°C for 30 seconds; 61°C for 30 seconds; 72°C for 30 seconds); and 72°C for 10 minutes. The primer sets used for the desired target regions were as follows: 5′-ATTGAAGTAATTAATTTAAAGGATAGTGTA-3′ (forward), 5′-AAAAACTACAAAC-CAAACCTTAAAC-3′ (reverse), for *NOX4*; 5′-TTTGTATGGAATTGTAAAATAATTGAGT-3′ (forward 1), 5′-CCTATAAAAAATCAACAAACAAATAACC-3′ (reverse 1), and 5′-AGTTATATTATTTTTTTGTTTTGAAAGT-3′ (forward 2), 5′-CAACCTACTTAAA-TCACACTCC-3′ (reverse 2), for *GCH1*. The PCR products were then separated and assessed on 1% agarose gels. The specific band products of the appropriate size were extracted from the gel with Gel-Out Concentrator (A&A Biotechnology). Purified PCR products were then A-tailed with Taq Polymerase (Thermo Fisher Scientific) and ligated into the pGEM-T Easy Prelinearized Vector System (Promega), which contains 3′-T overhangs. After transformation, recombinant clones were selected by blue/white screening on indicator plates. The insert presence in selected clones was confirmed by restriction enzyme digestion. The chosen constructs were sequenced using the Sanger method. The sequences obtained for the *NOX4* gene promoter were analyzed using BiQ Analyzer 3.0.

### Co-IP.

The co-IP procedure was performed as previously described (63). Briefly, harvested HDMECs were homogenized in precipitation lysis buffer (20 mM Tris, pH 8.0, 75 mM NaCl, 15 mM MgCl_2_, 1 mM EDTA, 0.5% Nonidet P-40, 10% glycerol, and protease inhibitor cocktail). Homogenates containing 500 μg proteins were incubated overnight at 4°C with 1 μg anti-eNOS antibody (BD Biosciences, 610297) and 20 μL prewashed protein A/G PLUS Agarose Beads (Santa Cruz Biotechnology). Precipitated protein complexes were washed 6 times with lysis buffer, resuspended in electrophoresis sample buffer, boiled for 10 minutes at 95°C, and then analyzed by immunoblotting using an antibody directed against HSP90α (BD Biosciences, 610419).

### Aldosterone assay.

Blood was collected from heparinized animals. After centrifugation, plasma samples were immediately stored at –80°C. The plasma was diluted 3 times in the calibration diluent RD5-69 prior to measurement. The aldosterone levels were determined using the Aldosterone Assay (R&D Systems) according to the manufacturer’s instructions (54).

### ELISA.

Urine was collected from the bladder after sacrifice and stored at –80^°^C. Mouse albumin (Abcam) and mouse NGAL (R&D Systems) ELISAs were performed according to the manufacturer’s protocols. Creatinine was measured using the Creatinine Parameter Assay Kit (R&D Systems). Albumin and NGAL levels were normalized to urinary creatinine levels.

### Luciferase reporter assay.

Transfections were performed using the Lipofectamine 2000 transfection reagent (Thermo Fisher Scientific) according to the manufacturer’s instructions. Briefly, 1 day before transfection, 1 × 10^5^ HEK293 cells were seeded in DMEM (Invitrogen, Thermo Fisher Scientific) with 10% FBS (Supplemental Table 3). Cells were 70%–90% confluent at the time of transfection. For transfection, the culture medium was changed to opti-MEM (Invitrogen, Thermo Fisher Scientific). Cells were transfected with NOX4 promoter – luciferase reporter plasmid DNA (GeneCopoeia HPRM35825-PG02-50; 0.1 μg) and *Renilla* plasmid DNA (0.05 μg, as a transfection control) mixed with Lipofectamine 2000. Four hours after transfection, the transfection media were replaced with DMEM, and the cells were treated with 100 nM docetaxel or vehicle. Cells were harvested 48 hours after transfection, and luciferase activity was measured using a Dual Luciferase reporter assay system (Promega, E1910).

### Data availability.

RNA-Seq raw data have been deposited in the NCBI’s Gene Expression Omnibus (GEO) database (GEO GSE202682).

### Statistics.

The results were analyzed using Statistica 13 or GraphPad Prism 6.0 and 9.0 (GraphPad Software). Continuous variables of clinical data were initially evaluated to check the normality of the distribution using the Shapiro-Wilk test, and then depending on the obtained distribution, the Mann-Whitney *U* test or Student’s *t* test was used. The χ^2^ test was used to compare the frequencies. Comparisons of 2 groups were performed using a 2-sided Student’s *t* test for paired or unpaired samples. Comparisons of more than 2 groups were performed using a 1- or 2-way ANOVA with Bonferroni’s or Tukey’s multiple-comparison correction. *P* values of less than 0.05 were considered significant. Overall *P* values for 2-way ANOVA are presented in Supplemental Table 1. Values are presented as the mean ± SEM. Paired, differential gene expression analysis from the RNA-Seq experiment was performed using the DESeq2 package in R, version 3.5.1 (64). Gene set enrichment analysis (GSEA) was performed with the fgsea (65) package in R and the Gene Ontology (Biological Process) set (version 6.2), using all transcripts with calculated test statistics.

### Study approval.

The study was conducted with the approval of the local bioethics committee of Jagiellonian University. Written informed consent was obtained from all patients. Animal studies were approved by the first and second Local Ethics Committees for Experiments on Animals in Cracow (approval nos. 20/2016 and 529/2021).

## Author contributions

TJG, DGH, TG, and PS conceived the study and designed experiments. PS, MS, RN, A Dikalova, SD, TPM, EJ, A Dziewulska, AMD, BS, WC, and JD conducted experiments and acquired data. PS and MS analyzed data. DHZ, JS, KG, PB, JK, JSG, and IL performed the clinical study and collected clinical data. TJG and PS wrote the manuscript. DGH, TG, JM, A Dobrzyn, FC, MCG, PM, MT, MS, and TPM helped write the manuscript and made intellectual contributions to it.

## Supplementary Material

Supplemental data

Supplemental table 4

Supplemental table 5

## Figures and Tables

**Table 1:**
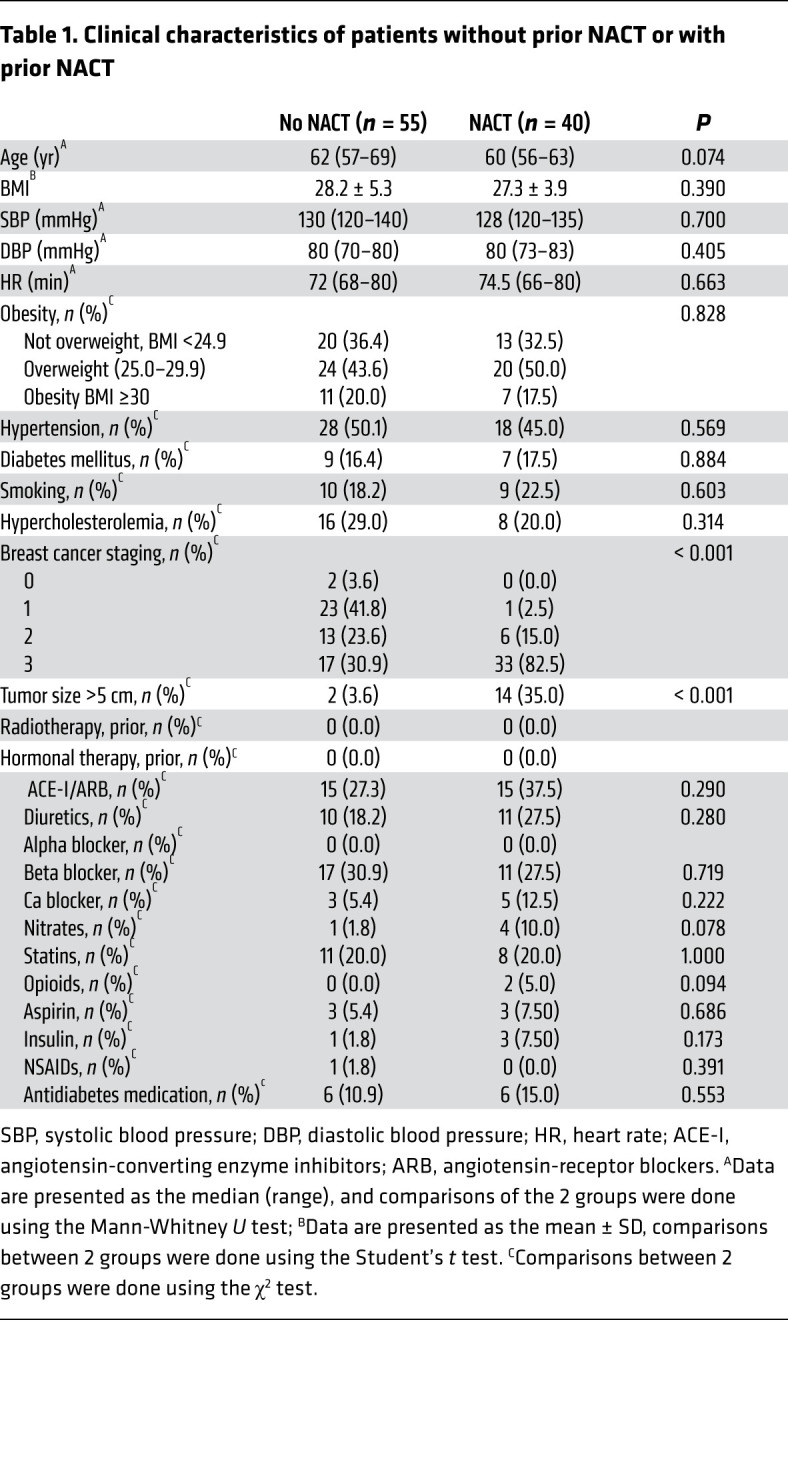
Clinical characteristics of patients without prior NACT or with prior NACT

## Version 1 05/26/2022

In-Press Preview

## Version 2 07/01/2022

Electronic publication
