# Supplementary material for: Breast cancer chemotherapy induces vascular dysfunction and hypertension through a NOX4-dependent mechanism
Source: J Clin Invest. 2022 Jul 1;132(13):e149117. doi: 10.1172/JCI149117 (PMC9246378; doi:10.1172/JCI149117)
Supplement: Supplemental data [file jci-132-149117-s126.pdf]

SUPPLEMENTAL MATERIALS

Breast Cancer Chemotherapy Induces Vascular Dysfunction and Hypertension through NOX4 dependent mechanism.

Piotr Szczepaniak[1,2,3], Mateusz Siedlinski[1,2], Diana Hodorowicz-Zaniewska[4], Ryszard Nosalski[1,2], Tomasz P. Mikolajczyk[1,2], Aneta M. Dobosz[5], Anna Dikalova[6], Sergey Dikalov[6], Joanna Streb[7], Katarzyna Gara[4], Pawel Basta[8], Jaroslaw Krolczyk[9], Joanna Sulicka-Grodzicka[10], Ewelina Jozefczuk[1], Anna Dziewulska[5], Blessy Saju[2], Iwona Laksa[7], Wei Chen[6], John Dormer[11], Maciej Tomaszewski[12,13], Pasquale Maffia[2,14,15], Marta Czesnikiewicz-Guzik[16,17], Filippo Crea[18], Agnieszka Dobrzyn[5], Javid Moslehi[19], Tomasz Grodzicki[9], David G. Harrison[6], Tomasz J. Guzik[1,2]

[1]Department of Medicine, Jagiellonian University Medical College, Krakow, Poland
[2]Institute of Cardiovascular and Medical Sciences, University of Glasgow, UK.
[3]Centre for Cardiovascular Science, Queen's Medical Research Institute, University of Edinburgh, UK.
[4]Breast Unit, Department of Surgery, Jagiellonian University Hospital, Krakow, Poland.
[5]Laboratory of Cell Signaling and Metabolic Disorders, Nencki Institute of Experimental Biology of Polish Academy of Sciences, Warsaw, Poland.
[6]Division of Clinical Pharmacology, Department of Medicine, Vanderbilt University, Nashville, TN.
[7]Department of Oncology, Jagiellonian University Medical College, Krakow, Poland.
[8]Department of Gynecology and Oncology, Jagiellonian University Medical College, Krakow, Poland.
[9]Department of Internal Medicine and Gerontology and Division of Oncology, Jagiellonian University Medical College, Krakow, Poland.
[10]Department of Rheumatology, Jagiellonian University Medical College, Krakow, Poland.
[11]Department of Cellular Pathology, University Hospitals of Leicester, Leicester, UK.
[12]Division of Cardiovascular Sciences, Faculty of Medicine, Biology and Health, University of Manchester, Manchester, UK.
[13]Division of Medicine and Manchester Academic Health Science Centre, Manchester University NHS Foundation Trust Manchester, Manchester, UK.
[14]Institute of Infection, Immunity and Inflammation, University of Glasgow, UK.
[15]Department of Pharmacy, School of Medicine and Surgery, University of Naples Federico II, Naples, Italy.
[16]Department of Periodontology, Preventive Dentistry and Oral Pathology, Krakow, Poland.
[17]Department of Periodontology and Oral Sciences Research Group, University of Glasgow Dental School, UK.
[18]Department of Cardiovascular and Thoracic Sciences, Catholic University of the Sacred Heart, Largo A. Gemelli, Rome, Italy.
[19]Section of Cardio-Oncology & Immunology, Division of Cardiology and the Cardiovascular Research Institute, University of California San Francisco, San Francisco, CA, USA.

Corresponding author:
Tomasz J Guzik MD PhD FRCP,
Institute of Cardiovascular and Medical Sciences,
University of Glasgow,
120 University Place,
Glasgow G12 8TA,
United Kingdom
Tel.+44(0)1413307590;
Email: tomasz.guzik@glasgow.ac.uk

SUPPLEMENTAL DATA

A

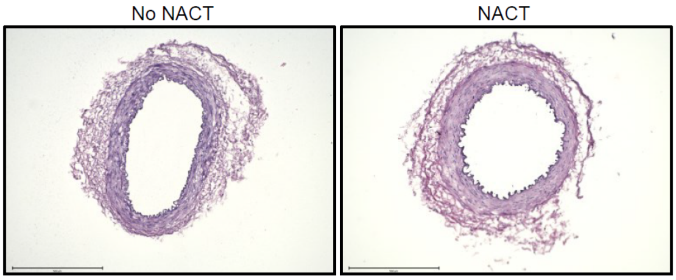

	No NACT	NACT	P
Vessel diameter (µm)	842.1±162.3	850.9±149.0	0.9
Wall thickness (µm)	155.7±39.3	160.4±21.6	0.8

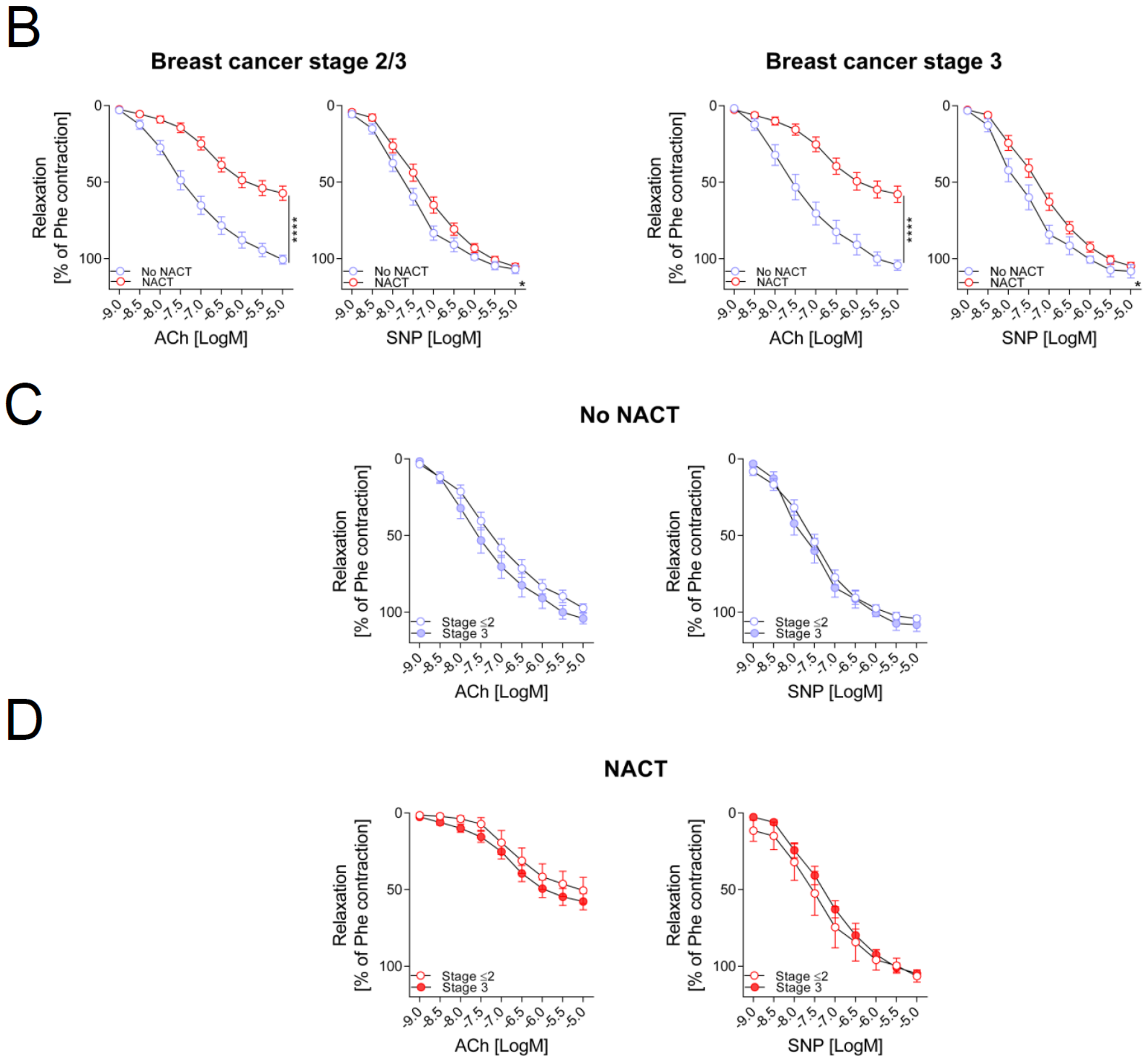

Supplemental Figure 1: Comparison of blood vessel morphology in NACT and No NACT and effects of the stage of breast cancer on endothelial dysfunction. (A) Vascular

morphology of studied vessels shown in representative H&E histograms (top) and quantification of vascular morphology (bottom) (n=5 per group). Scale bar, 500 μm. Data are expressed as mean ± s.e.m.; p-value - two-tailed unpaired t-test. (**B**) Average endothelium-dependent vasorelaxation curves to ACh (1 nM to 10 μM) and endothelium-independent vasorelaxations in response to SNP (1 nM to 10 μM) in vessels from patients without NACT (No NACT; n=30) and from patients who underwent NACT (NACT; n=39) with stages 2 and 3 of breast cancer (left) and in vessels from patients without NACT (No NACT; n=17) and from patients who underwent NACT (NACT; n=33) with stage 3 of breast cancer (right). Data are expressed as mean ± s.e.m. ****$P<0.0001$, *$P<0.05$ vs. No NACT. Data were analyzed with repeated-measures ANOVA. (**C-D**) Comparison of endothelium-dependent vasorelaxations curves to ACh (1 nM to 10 μM) (left) and to SNP (1 nM to 10 μM) (right) breast cancer stage ≤2 with breast cancer stage 3 in vessels (**C**) from patients without NACT (No NACT; left; n=17 vs 38) and (**D**) from patients who underwent NACT (NACT; right; n=7 vs 33). Data are expressed as mean ± s.e.m. Data were analyzed with repeated measures ANOVA.

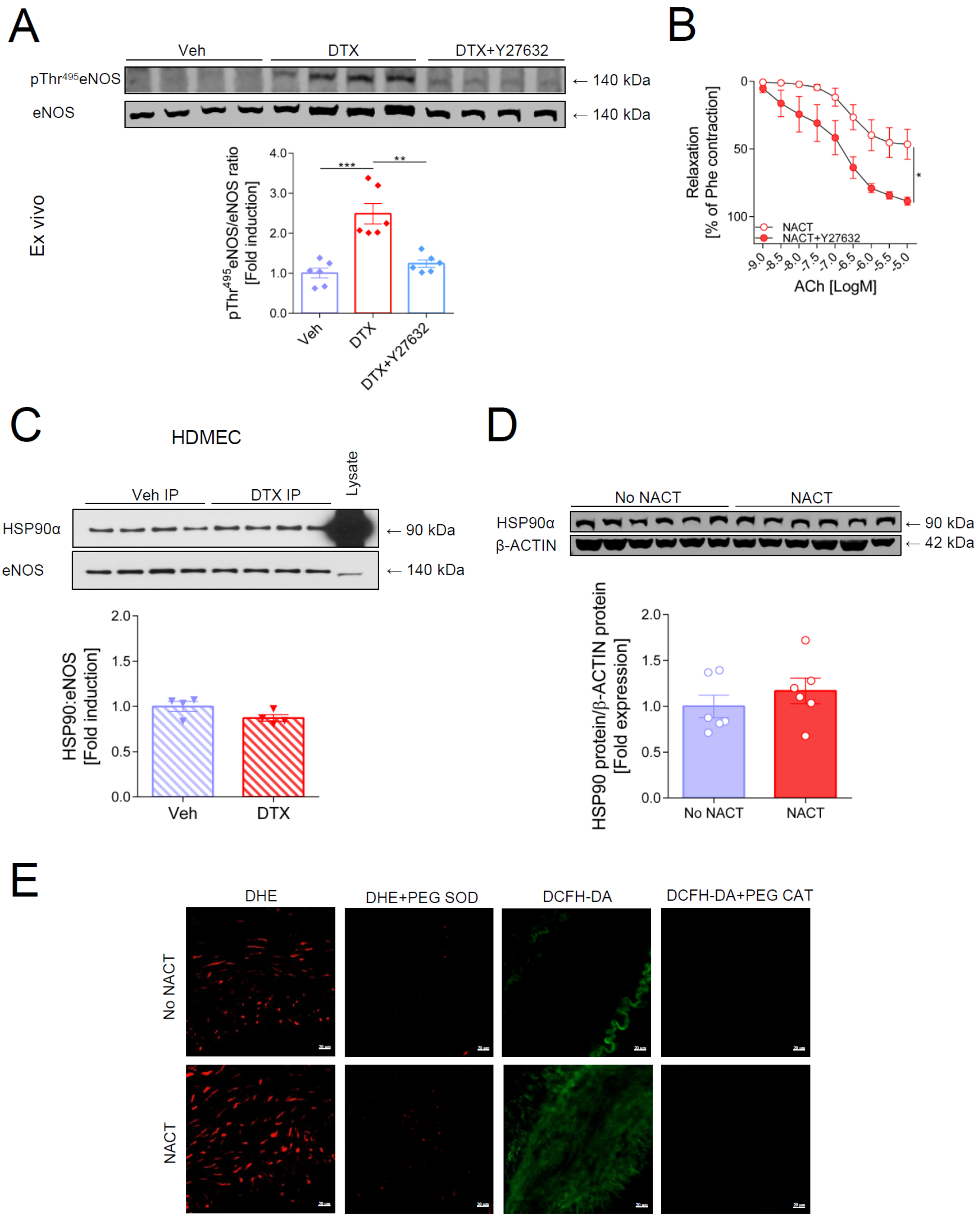

Supplemental Figure 2: Effects of breast cancer neoadjuvant chemotherapy on the regulation of endothelial nitric oxide synthase and the role of oxidative stress in NACT-induced endothelial dysfunction. **(A)** Effects of DTX (100 nM) on eNOS at Thr495

phosphorylation in NACT-naive vessels after 24-hour organ culture and the modulating effect of Y27632 (5 µM) (n=6 per group). Densitometric analysis of proteins normalized to the expression of total eNOS. Immunoblots are shown from one experiment (upper). Data are derived from two independent experiments and expressed as mean ± s.e.m. ***P<0.001 vs. Veh, **P<0.01 vs. DTX. Data were analyzed with one-way ANOVA and Tukey's test. (**B**) Effect of Y27632 (5 µM) on endothelium-dependent vasorelaxation to acetylcholine (ACh) in vessels from patients with prior neoadjuvant chemotherapy (NACT) (n=7/group). Data shown as mean ± s.e.m; *P<0.05 vs NACT by repeated measures ANOVA. (**C**) Effects of DTX (100 nM) on association between eNOS and HSP90α in Human Dermal Microvascular Endothelial Cells (HDMEC) (n=4/group). Data are expressed as mean ± s.e.m. Two-tailed unpaired t-test. (**D**) Determination of HSP90α in vessels from patients with and without prior neoadjuvant chemotherapy (NACT) with densitometric analysis (n=6/group). Data are expressed as mean ± s.e.m. Two-tailed unpaired t-test. (**E**) High-power microphotographs of fluorescence for detection of superoxide (dihydroethidium; DHE; 10 µM- magnification of images in Figure 3F) and H_2O_2 production (2',7'-dichlorodihydrofluorescein diacetate; DCFH-DA; 10 µM) in vessel sections from patients with/without prior NACT. PEG-superoxide dismutase (PEG SOD; 500 U/ml) and PEG-catalase (PEG CAT; 500 U/ml) were used to show signal specificity for superoxide and H_2O_2 respectively (representative of n=5/group). Scale bar, 20 µm.

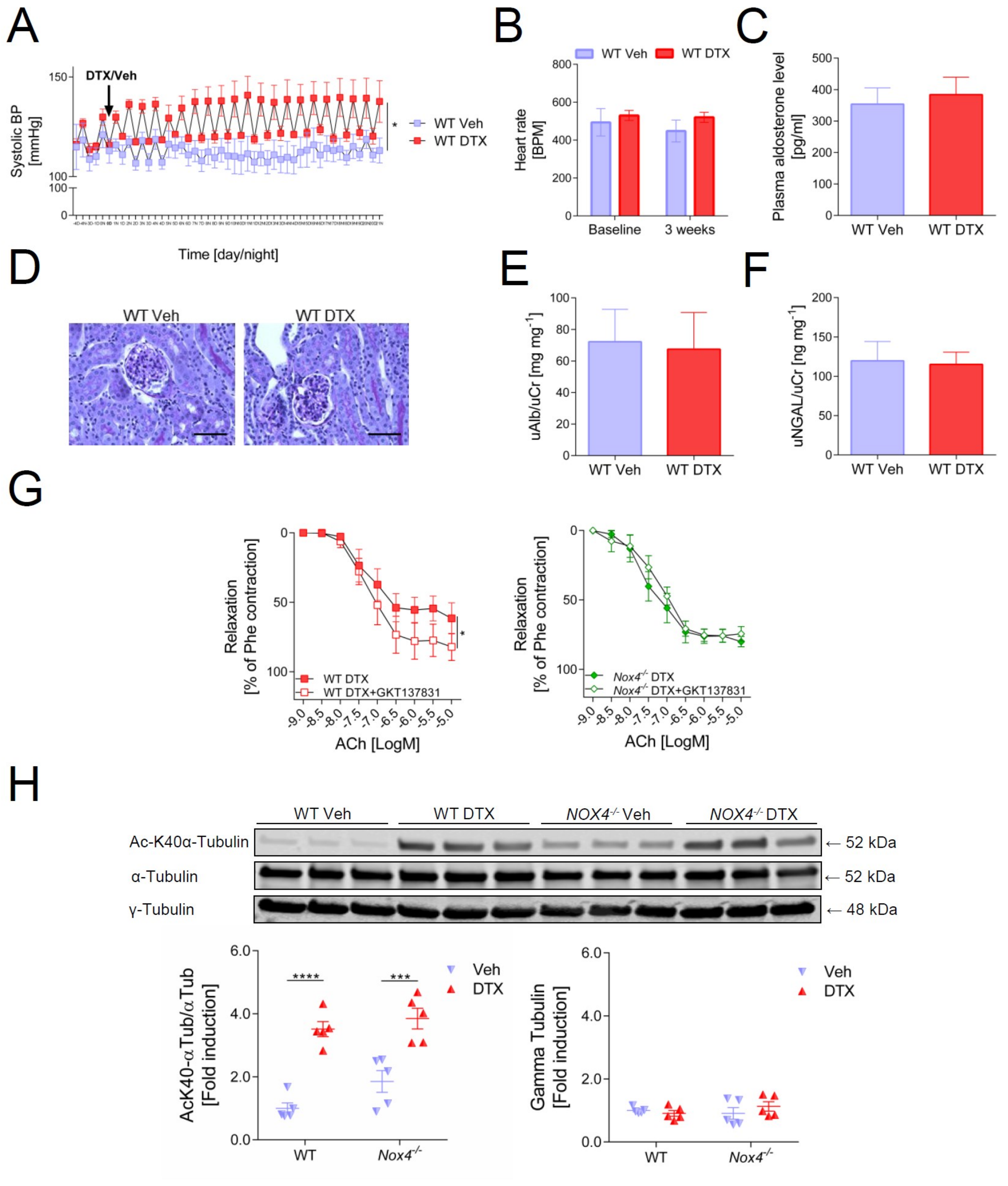

Supplemental Figure 3. Effects of docetaxel on blood pressure, renal function, microtubules and determination of the role of Nox4 in endothelial dysfunction induced by docetaxel. (**A**) Systolic blood pressure (SBP) measured using telemetry in wild-type (WT)

mice treated with docetaxel (DTX) or placebo (n=4 per group). Data are expressed as mean ± s.e.m. *P<0.05 vs WT. Data were analyzed with repeated measures ANOVA. (**B**) Heart rate measured using telemetry in WT mice treated with DTX or placebo (n=4 per group). Data are expressed as mean ± s.e.m. Data were analyzed with two-way ANOVA with Tukey's test. **(C)** Aldosterone levels in WT mice with DTX or placebo (n=7/group). Data are expressed as mean ± s.e.m. Data were analyzed with a two-tailed unpaired t-test. **(D)** Representative images of kidney staining with periodic acid - Schiff (PAS) in WT mice treated with DTX or placebo (n=6-7/group). Scale bar- 60 μm. **(E)** Urine albumin (uAlb) level in WT mice with DTX or placebo (n=6/group). Data are expressed as mean ± s.e.m. Data were normalized with urine creatinine (uCr) level. Data were analyzed with a two-tailed unpaired t-test. **(F)** Urine NGAL level in WT mice with DTX or placebo (n=6/group). Data are expressed as mean ± s.e.m. Data were normalized with urine creatinine (uCr) level. Data were analyzed with two-tailed unpaired t-test. (**G**) Endothelium dependent vasorelaxation in response to acetylcholine (ACh; 1 nM to 10 μM) after preincubation with GKT137831 (10 μM) in WT DTX mice (n=7 per group; left) and *Nox4*$^{-/-}$DTX mice (n=7 per group; right). Data are expressed as mean ± s.e.m. *P<0.05 vs. WT. Data were analyzed with repeated measures ANOVA. **(H)** Effect of DTX on Ack40-α-Tubulin and γ-Tubulin in *Nox4*$^{-/-}$ and WT mice treated with DTX or placebo (n=5/group). Densitometric analysis of Ack40-α-Tubulin normalized to the expression of total α-Tubulin. Immunoblots are shown from one experiment (upper). Data are derived from two independent experiments and expressed as mean ± s.e.m. ****P<0.0001 vs. WT DTX; ***P<0.001 vs. *Nox4*$^{-/-}$DTX. Data were analyzed with two-way ANOVA and Bonferroni test.

Supplemental Table 1. Overall P values for two-way ANOVA.

**Overall P values for two-way ANOVA**			
Figure 1F for ACh	$P^{response}<0.0001$	$P^{group}=0.0475$	$P^{response\ x\ group}=0.4781$
Figure 1F for SNP	$P^{response}<0.0001$	$P^{group}=0.8787$	$P^{response\ x\ group}=0.9955$
Figure 2A for ACh	$P^{response}<0.0001$	$P^{group}=0.0003$	$P^{response\ x\ group}<0.0001$
Figure 6C for SBP	$P^{time}=0.0001$	$P^{group}=0.2436$	$P^{time\ x\ group}=0.0096$
Figure 6D for ACh	$P^{response}<0.0001$	$P^{group}=0.0005$	$P^{response\ x\ group}<0.0001$
Figure 6D for SNP	$P^{response}<0.0001$	$P^{group}=0.3692$	$P^{response\ x\ group}=0.9819$
Figure 6E for H_2O_2	$P^{genotype}=0.0015$	$P^{DTX}=0.0109$	$P^{genotype\ x\ DTX}=0.1143$
Figure 6F for LGCL	$P^{genotype}=0.1755$	$P^{DTX}=0.0012$	$P^{genotype\ x\ DTX}=0.1289$
Figure 6H for *Nox4*	$P^{genotype}<0.0001$	$P^{DTX}=0.0198$	$P^{genotype\ x\ DTX}=0.0198$
Figure 6I for Thr 495	$P^{genotype}=0.1133$	$P^{DTX}=0.0071$	$P^{genotype\ x\ DTX}=0.0007$
Figure 7B for SBP	$P^{time}=0.0261$	$P^{group}=0.0146$	$P^{time\ x\ group}=0.0198$
Figure 7C for ACh	$P^{response}<0.0001$	$P^{group}=0.0045$	$P^{response\ x\ group}=0.0062$
Figure 7C for SNP	$P^{response}<0.0001$	$P^{group}=0.1409$	$P^{response\ x\ group}=0.0653$
Figure 7E for SBP	$P^{time}=0.3375$	$P^{group}=0.0120$	$P^{time\ x\ group}=0.0281$
Figure 7F for ACh	$P^{response}<0.0001$	$P^{group}=0.0300$	$P^{response\ x\ group}=0.0139$
Figure 7F for SNP	$P^{response}<0.0001$	$P^{group}=0.5048$	$P^{response\ x\ group}=0.1105$
Supplemental Figure 3B for HR	$P^{time}=0.0554$	$P^{group}=0.4657$	$P^{time\ x\ group}=0.1749$
Supplemental Figure 3H for Ack40-Tub	$P^{genotype}=0.0505$	$P^{DTX}<0.0001$	$P^{genotype\ x\ DTX}=0.3678$
Supplemental Figure 3H for gamma-Tub	$P^{genotype}=0.6167$	$P^{DTX}=0.6263$	$P^{genotype\ x\ DTX}=0.2384$

Supplemental Table 2. Antibodies.

Target antigen	Source	Catalog number
β-ACTIN	Abcam	ab8226
phospho-eNOS (Thr495)	BD Biosciences	612707
phospho-eNOS (Ser1177)	BD Biosciences	612393
eNOS	BD Biosciences	610297
NOX1	Sigma-Aldrich	HPA035299
NOX2	Abcam	ab129068
NOX4	Abcam	ab109225
NOX5	Sigma-Aldrich	SAB2501641
α-Tubulin	Cell Signaling	2125S
acetyl-α-Tubulin	Cell Signaling	5335S
gamma-Tubulin	Sigma-Aldrich	T6557
HSP90α	BD Biosciences	610419
CD31	Abcam	ab24590
α-SMA	Sigma-Aldrich	A5228

Supplemental Table 3. Cell lines.

Cell line	Source
Human Dermal Microvascular Endothelial Cells (HDMEC)	PromoCell
Human Aortic Smooth Muscle Cells (HASMC)	Thermo Fisher
HEK 293	ATCC
